# Unraveling aryl peroxide chemistry to enrich optical properties of single-walled carbon nanotubes†

**DOI:** 10.1039/d4sc04785k

**Published:** 2024-12-11

**Authors:** Patrycja Taborowska, Andrzej Dzienia, Dawid Janas

**Affiliations:** a Department of Chemistry, Silesian University of Technology B. Krzywoustego 4 44-100 Gliwice Poland Andrzej.Dzienia@polsl.pl Dawid.Janas@polsl.pl

## Abstract

Harnessing the unique optical properties of chirality-enriched single-walled carbon nanotubes (SWCNTs) is the key to unlocking the application of SWCNTs in photonics. Recently, it has been discovered that chemical modification of SWCNTs greatly increases their potential in this context. Despite the dynamic progress in this area, the mechanism of the chemical modification of SWCNTs and the impact of the reaction conditions on the properties of the obtained functional nanomaterials remain unclear. In this study, we demonstrate how the reaction environment influences the observed fluorescence pattern of SWCNTs after modification with benzoyloxy radicals generated *in situ*. The obtained results reveal that each diacyl peroxide molecule can generate either one or two radicals by two different mechanisms, *i.e.*, induced or spontaneous decomposition. Through proper selection of the reactant concentration, process temperature, and solvent, we were able to activate one or both radical decay pathways. In addition, the choice of a solvent, such as tetrahydrofuran or acetonitrile, allowed drastic changes in the functionalization process. Consequently, the SWCNT surface was grafted with functional groups *via* C–C bonds using radicals derived from the solvent molecules instead of attaching an aromatic moiety from the reactant present in the system through the expected C–O linkage. Verification of the structure of the chemically bound functional groups through hydrolysis opens the route to further modification of SWCNT surfaces using the labile ester connection. By gaining a better understanding of the emergence and behavior of the generated radicals, we demonstrate the possibility of controlling the density of introduced defects, as well as the selectivity of the functionalization process. The identification of the underlying chemical pathways responsible for the functionalization of SWCNTs paves the way for the design of precise methods of SWCNT modification to adjust their photonic characteristics for specific applications.

## Abstract 2

Harnessing the unique optical properties of chirality-enriched single-walled carbon nanotubes (SWCNTs) is the key to unlocking the application of SWCNTs in photonics.

## Graphical abstract

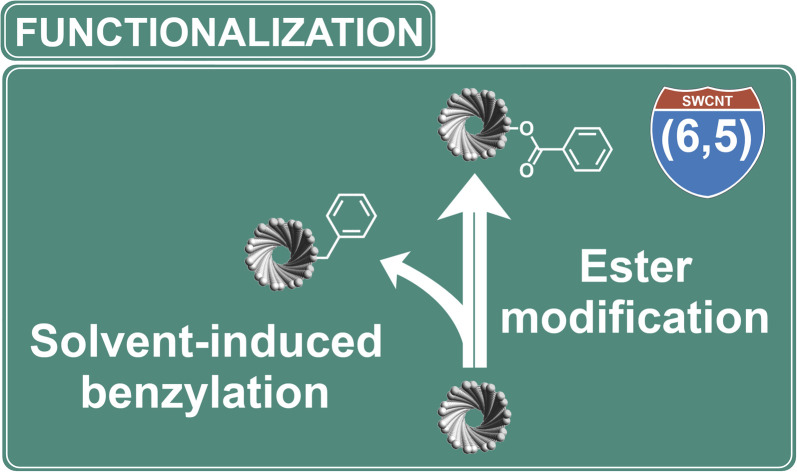

## 1. Introduction

Semiconducting single-walled carbon nanotubes (s-SWCNTs) are attractive candidates for modern applications in photonics because they exhibit narrow-band emission in a range spanning from the visible to near-infrared (NIR).^1,2^ The chirality of an SWCNT dictates its bandgap, and thus also the spectral positions of the optical transitions E_11_, E_22_, and E_33_, which are observed in the absorption and photoluminescence (PL) emission spectra^3^ (typically, the emission at E_11_ is observed upon excitation at E_22_). However, the spectra of the as-synthesized SWCNTs are usually quite complex because the material consists of at least several chiral species. Defined optical characteristics, which are necessary for applications, can be achieved with post-synthetic sorting,^4^ among which conjugated polymer extraction (CPE) is one of the most promising techniques. It allows for quick isolation of single-chirality SWCNTs with purity levels exceeding 90% and yields up to 10%, demonstrating a potential for large-scale production.^5^ SWCNT material purified *via* CPE can be subsequently modified chemically^2,6^ or physically^7^ to fine-tune its optical properties.

The photoluminescence quantum yield (PLQY) from pristine semiconducting SWCNT is unsatisfactory (<1% measured in aqueous dispersions^8^ and up to 5.0% for CPE-isolated SWCNTs^9^), but can be improved by the covalent bonding of certain functional groups to the surface of SWCNTs. This processing introduces synthetic sp^3^ defects (also known as Organic Color Centers^10^) into an otherwise sp^2^ hybridized arrangement of carbon atoms, the presence of which may be highly beneficial. Such lattice imperfections increase the probability of the radiative recombination of excitons by generating a new state localized within the continuum bands of the SWCNT host. As a consequence, the PLQY can be substantially increased.^11^ The newly produced emission peak, referred to as 
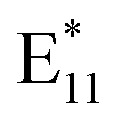
, is red-shifted from the E_11_ optical transition by 120–160 nm.^8,10,12^ Further red-shifted (by *ca.* 260 nm) peaks (
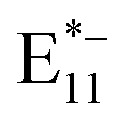
, 
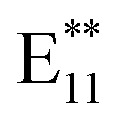
, or 
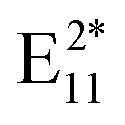
 (ref. 2, 9, and 13)) emerge, *e.g.*, when divalent functional groups are attached to the surface of SWCNTs^6,14^ or upon an appropriately high degree of SWCNT modification. A wide variety of methods for the functionalization of SWCNTs have been developed, where SWCNTs in the form of dispersions or films are subjected to oxidation, alkylation, or arylation, a more elaborative description of which can be found in review articles on the topic.^1,2,8^ Lately, SWCNT functionalization tactics conducted in organic solvents have been the focal point of the scientific community, as they allow the creation of suspensions of SWCNTs with improved optical performance, which can be easily and, most importantly, directly deposited on various substrates due to the typically high volatility of such solvents.^12^ Of the wide range of reactants available for this processing, the most popular are various diazonium salts, but many structures can also be attached by reductive alkylation with aryl or alkyl halides in organic solvents.^15^ The former approach was initially used in water because it ensures good dispersity of these highly polar functionalization agents.^8,13,16–19^ However, the processing can be conducted in organic media as well, for instance, upon the addition of a polar co-solvent and phase-transfer agent.^20^ Unfortunately, in the diazonium approach, the reactions' protocols often require keeping the dispersion in the dark for over 12 hours (ref. 20) or even several days,^8,13,21^ while functional groups slowly bond to the SWCNT surface. The reaction rate can be increased by light irradiation,^19^ which proved effective for the covalent modification of SWCNTs with 2-iodoaniline^9^ and sodium hypochlorite.^22^ In other approaches, sonochemical treatment^23^ and high temperatures^24^ also substantially reduced the reaction time.

Organic peroxides were one of the first reagents to be used to modify SWCNTs by introducing new covalent bonds.^25–28^ This process of functionalization has previously been widely studied for the sake of improving CNT dispersibility,^26,29^ for aligning SWCNTs in polypropylene/SWCNT composite fibers to boost their mechanical properties,^30^ or for the design of radical scavengers from SWCNTs.^31^ Regarding the latter objective, the catalytic effect of CNTs on the decomposition of benzoyl peroxide (BPO) in ethanol and other polar organic solvents was recently reported.^32^ Unfortunately, despite its potential merits, the possibility of using this functionalization route to enhance the photonic characteristics of SWCNTs by the incorporation of a small number of defects into SWCNTs remains largely unexplored.

The lack of progress on this front likely results from the complexity of radical chemistry. Understanding the kinetics of the functionalization process and determining the structure of the attached groups are challenging because radicals from acyl peroxides, with BPO being the most significant example, can be generated through various mechanisms. Additionally, their formation can be mediated by solvent molecules, which stabilize the transition state during O–O bond breakage. Certain solvent molecules can also donate a hydrogen atom to the benzoyloxy radical, resulting in new radicals' formation through a mechanism known as chain transfer. Typically, peroxides generate two radicals by spontaneous unimolecular homolytic cleavage of the O–O bond,^33^ which allows for two types of SWCNT functionalization: monovalent R-SWCNT (low degree of functionalization) and divalent R-SWCNT-R (high degree of functionalization).^34^ While this provides an advantage in obtaining a substantially shifted 
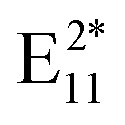
 peak (positioned at *ca.* 1260 nm for modified (6,5) SWCNTs), it also presents a challenge in controlling its selective generation relative to 
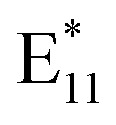
 (located at approximately 1160 nm for this chirality). Concomitantly, radicals present in the system can also participate in the decomposition of BPO, through solvent-induced or radical-induced bimolecular decomposition, which generates only one radical, along with unreactive compounds, such as phenyl benzoate.^33,35,36^

While the abovementioned studies are undoubtedly insightful for understanding the nature of peroxide radicals, they are insufficient to comprehend the mechanism of SWCNT functionalization with radical reactants such as BPO. Given the rich and diverse nature of radical chemistry,^26,27^ it is of utmost importance to interpret how such compounds may be used to enrich the optical properties of SWCNTs. For instance, it is essential to understand how the process conditions affect the kinetics of SWCNT functionalization as well as the pattern of the resulting fluorescence spectrum. To this end, we explored the influence of a broad range (*T*_func._ = 40 to 100 °C) of reaction temperatures, the impact of processing conditions, and also considered solvent selection. These factors were investigated in detail to develop a precise tool for modulating the PL of SWCNTs, harnessing the potential of radical reactions. Our studies have shown that, depending on the reaction conditions and system composition, it is possible to tune the SWCNT functionalization selectivity to form the desired types and morphologies of defects, and, consequently, tailor the PL characteristics of the material. We believe that the results obtained will enable wider exploitation of the advantages and possibilities of radical chemistry, laying the foundation for the straightforward introduction of more complex organic structures onto the surface of SWCNTs.

## 2. Experimental

### 2.1 Materials

All chemical reagents, *i.e.*, 9,9-dioctyl-2,7-dibromofluorene (Angene, cat. number: AG002BU7, CAS: 198964-46-4, purity: 98%), 9,9-dioctylfluorene-2,7-bis(boronic acid pinacol ester) (Angene, cat. number: AG0034EZ, CAS: 196207-58-6, purity: 98%), Aliquat 336 TG (Alfa Aesar, cat. number: A17247, CAS: 63393-96-4, purity: N/A), tetrakis(triphenylphosphine)palladium – Pd(PPh_3_)_4_ (Apollo Scientific, cat. number: OR4225, CAS: 14221-01-3, purity: >99%), benzoyl chloride (Sigma-Aldrich, cat. number: 259950, CAS: 98-88-4, purity: 99%), sodium hydroxide (Chempur, cat. number: 118109252, CAS: 1310-73-2, purity for analysis), hydrogen peroxide 30% (Chempur, cat. number: 118851934, CAS: 7722-84-1, purity for analysis), benzoyl peroxide (VWR Chemicals, cat. number: L13174.30, CAS: 94-36-0, purity: 97% (dry weight), moistened with 25% water), and tetramethylammonium hydroxide (TMAH, Thermo Scientific, cat. number: 11327368, CAS: 75-59-2, electronic grade, purity: >99.9%), and solvents, *i.e.*, toluene (Alfa Aesar, cat. number: 19376.K2, CAS: 108-88-3, spectrophotometric grade, purity: >99.7%), toluene-d_8_ (Deutero, cat. number: 01502, CAS: 2037-26-5, purity: 99%), hexane (Alfa Aesar, cat. number: L09938.AK, CAS: 110-54-3, purity: 99%), cyclohexane (Alfa Aesar, cat. number: A16070.0J, CAS: 110-82-7, purity: 99%), decahydronaphthalene (decalin, Alfa Aesar, cat. number: A13883.AP, CAS: 91-17-8, purity: 98%, *cis* + *trans*), tetrahydronaphthalene (tetralin, Fisher Chemical, cat. number: T/0850/08, CAS: 119-64-2, purity: >97%), tetrahydrofuran (THF, Alfa Aesar, cat. number: L13304.AP, CAS: 109-99-9, purity: 99%), acetonitrile (Alfa Aesar, cat. number: A19862.AP, CAS: 75-05-8, purity: >99%), diphenyl ether (Alfa Aesar, cat. number: A15791.36, CAS: 101-84-8, purity: 99%), *o*-xylene (Alfa Aesar, cat. number: A11358.AP, CAS: 95-47-6, purity: 99%), and 1,2-dichlorobenzene (DCB, Fisher Chemical, cat. number: D/1600/15, CAS: 95-50-1, purity: extra pure), were used as supplied, without additional purification or drying unless stated otherwise. The study was carried out using (6,5)-enriched CoMoCAT SG65i SWCNTs (Sigma-Aldrich, lot: MKCM5514, purity: 95% – carbon basis).

### 2.2 Standard extraction of SWCNTs

In a typical suspension process, 6 mg of PFO-BPy were dissolved in 5 mL of toluene. The solution was transferred to a 19 mL glass vial with 1.5 mg of pre-weighed SWCNTs. The mixture was homogenized in an ice-cooled bath sonicator (POLSONIC, SONIC-2, 250 W) for 10 minutes. Tip sonication was then carried out (Hielscher UP200St ultrasonic generator, equipped with a *Ø* 7 mm tip) at a power of 30 W for 8 min, while the mixture was cooled in an ice bath to keep a temperature of about 5 °C. After sonication, the thick suspension was transferred to a 15 mL conical tube and centrifuged at 10 000 rpm (15 314 g) for 3 minutes to remove the bundled SWCNTs and polymer aggregates. Finally, 90% of the supernatant was transferred to a fresh vial for characterization.

### 2.3 Thermal functionalization

0.5 mL of (6,5) SWCNTs derived from standard extraction with PFO-BPy were diluted to 0.3 cm^−1^ (E_11(6,5)_ optical absorbance peak maximum; unless stated otherwise). BPO was dissolved in toluene (0.5 mL) to achieve the necessary concentration. The indicated components were then combined in a glass vial and immersed in a stirred hot bath kept at 40, 70, 85, or 100 °C. After an hour, the samples were removed, cooled down to room temperature, and characterized. In the case of the samples chemically modified under inert conditions, the SWCNTs dispersed with polymer and the dissolved BPO were flushed with argon. Firstly, both components were bubbled with argon for 15 minutes separately; they were then mixed, and the bubbling was repeated for an additional 5 minutes prior to their immersion in a preheated bath. In the case of the samples of reduced moisture content, molecular sieves (4 Å) were added to the BPO solution, which was kept in a fridge overnight prior to the reaction to allow the water molecules to be adsorbed. The samples with intentional addition of H_2_O were prepared by adding 20 μL of deionized water to the 0.5 mL of BPO solution. All other modifications to the primary procedure are described in the text, figure captions or ESI.†

### 2.4 Characterization

Optical absorption spectra were recorded (immediately after centrifugation) with a Hitachi U-2910 spectrophotometer using a 5 mm pathlength quartz cuvette, with a cuvette containing pure toluene being placed in the reference channel. The recorded data were baseline-corrected and deconvoluted using PTF Fit software,^37^ as described by us previously,^38^ to quantify the concentrations of individual SWCNTs. The data were normalized to the peak of the highest intensity E_11_ transition whenever necessary for comparison of chirality distribution. The chiral purity (*p*) was expressed as the ratio of the concentration of a particular chirality (*n*, *m*) to the sum of concentrations of all the SWCNTs extracted in a specific experiment. PL excitation–emission maps were collected with a ClaIR plate reader (Photonetc. Inc.) equipped with an EVO HP EU-4 supercontinuum laser (NKT Photonics) and an LLTF Contrast bandpass filter (NKT Photonics) for excitation in the range 460–900 nm with emission measured from 947 to 1650 nm. For PL measurements, SWCNTs were diluted so that their concentration in the measured samples was low, *i.e.*, 0.27 μg mL^−1^ (optical density of 0.15 in the case of non-functionalized (6,5) chirality) to avoid the inner-filter effect.^39^ The PL spectra were extracted from the PL excitation–emission maps for the 574 nm excitation wavelength, normalized to the E_11_ or 
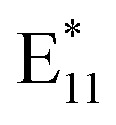
 intensity for spectral shape comparison, and fitted with a set of Voigt functions using self-developed Python scripts.

A detailed description of other apparatus and techniques used to carry out the investigations described, *i.e.*, gel permeation chromatography (GPC), Karl Fischer titration, ^1^H NMR, UV-VIS-NIR and Raman spectroscopy, is provided in the ESI.†

## 3. Results and discussion

**Fig. 1:**
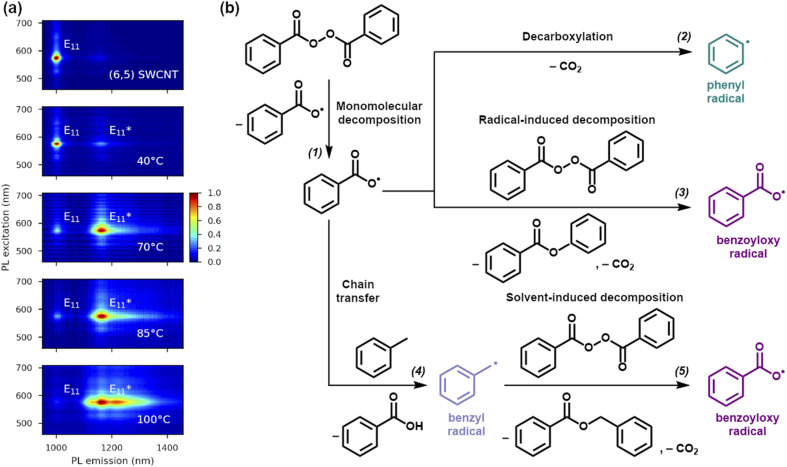
(a) PL excitation–emission maps of pristine (6,5) SWCNT dispersion with only one E_11_ peak and the dispersion treated for 1 hour with 15.0 mM BPO at 40, 70, 85, and 100 °C showing the emergence of 
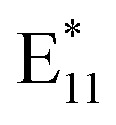
 defect peaks. (b) Schemes showing BPO decay reactions in toluene solution:^34,36,42,43^ (1) spontaneous thermal decomposition, (2) decarboxylation process of benzoyloxy radical, (3) benzoyloxy radical-induced decomposition of BPO, (4) chain transfer to solvent molecule, and (5) solvent-induced decomposition of BPO.

This study was based on the most popular chirality, *i.e.*, (6,5), to make the results readily comparable to future studies. This chirality was obtained in toluene by CPE using self-synthesized PFO-BPy. The structure and macromolecular characteristics were obtained by ^1^H NMR and GPC (Fig. S1†). Fig. 1a shows the PL map of pristine (6,5) SWCNTs, for which the E_11_ peak was found at 1001 nm, in agreement with previous studies for this chirality dispersed in toluene.^24,40^ The received purified dispersion of SWCNTs, after simple concentration adjustment, was reacted with BPO at various temperatures for 1 hour to create luminescent sp^3^ defects (Fig. 1a). The increase in reaction temperature promoted the emergence of new emissive states, which substantially changed the PL characteristics of the material. Interestingly, more than one defect-induced peak was registered after treating SWCNTs with BPO at 100 °C, revealing that the process temperature may determine the types of attached functional groups (*via* either oxygen or carbon linker) or their morphology (*ortho* L30, L-30, L90 and *para* L30, L-30, L90 positions).^9,17^

At this point, the thermally initiated peroxide decomposition needs to be discussed before interpreting these results. The decomposition of peroxides to radicals is a multifaceted process involving various kinetic and thermodynamic aspects.^33,35,41–43^ Considering the paramount influence of temperature on the initiation of peroxide decomposition into radicals, and subsequently on their concentration, diffusion processes, and the probability of recombination, our initial investigation focused on the analysis of SWCNT functionalization as a function of the reaction temperature. According to the literature, the self-accelerating decomposition temperature (SADT) determined for BPO is approximately 72–74 °C.^44^ Once this is reached, the decomposition of the O–O bonds occurs, and radicals are formed through a self-sustaining cascade of reactions.^45^ However, the value of the SADT strongly depends on the measurement methodology and the presence of solvents or stabilizers.^46^ Thus, BPO can be spontaneously decomposed into radicals even at much lower temperatures, especially in solution, as the process may be induced by small amounts of free radicals, contamination with acids, bases, or metals, and light irradiation.^47^ For example, reported SADTs for BPO in toluene are much lower, reaching values of just 28–30 °C.^45^

The typical monomolecular BPO homolytic scission (Fig. 1b – reaction (1)) produces two radicals, which can be attached to the SWCNT surface.^48^ This pathway dominates at low radical concentrations (*i.e.*, effective radical absorption) and high temperatures.^33^ It is important to note that, in the absence of radical absorbers (inhibitors), the benzoyloxy radicals are prone to decarboxylation into phenyl radicals (Fig. 1b – reaction (2)).^49^ Increasing the concentration of both types of radicals can also induce the decomposition of the BPO molecule *via* a mechanism referred to as induced bimolecular decomposition. This leads to the formation of only one radical (Fig. 1b – reactions (3) and (5)), and unreactive residues, such as phenyl benzoate and carbon dioxide.^33^ Furthermore, secondary benzyl radicals can be formed by the attack of the primary benzoyloxy radical on the solvent molecule and hydrogen atom abstraction, *e.g.*, from toluene (Fig. 1b – reaction (4)).^49^ This process is called a chain transfer reaction and is pronounced when acidic hydrogen atoms coming from alcohols, amines, or ethers (*e.g.*, diethyl ether, dioxane, or tetrahydrofuran) are present in the system.^50^ The abovementioned literature-based findings were confirmed by studying BPO decay by ^1^H NMR in toluene-d_8_, which verified its efficient degradation at 100 °C (Fig. S4–S7†) for two BPO concentrations. The experiments allowed tracking of the kinetics of the decomposition process (Fig. S8, Table S1†). In addition, ^1^H NMR measurements positively verified the thermal stability of PFO-BPy as well as its potential inertness towards the generated radicals under the experimental conditions explored (Fig. S9 and S10†). Besides, PFO-BPy did not seem to impact the type of radicals generated from BPO (Fig. S11–S13†).

The impact of a broad range of reaction temperatures, below, close to and above the SADT of BPO, *i.e.*, from 40 to 100 °C, on the PL of (6,5) SWCNTs is shown in Fig. 1a. For the same reaction time (1 hour) and BPO concentration (15 mM), various densities of the defects implanted into the SWCNTs were noted depending on the treatment temperature. At 70 °C, the 
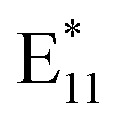
 peak at 1158 nm became dominant over the native E_11_ transition, the former being red-shifted by 163 meV from the E_11_. This value, referred to as the optical trap depth, was larger than previously reported values for oxidized, alkylated, or arylated, as well as alkyl-carboxylated SWCNTs^8,11,51–53^ measured in water or polar organic solvents. Notably, when comparing peak positions for particular structures attached to the surface of SWCNTs, it is important to take into account at least 20 nm solvatochromism between measurements carried out in water with a surfactant, compared to those taken with the SWCNTs suspended in organic solvents, *e.g.*, toluene, and the effect of wrapping with the conjugated polymer.^38^ Therefore, although signals at *ca.* 1158 nm originating from sp^3^ defects have already been reported in the literature,^54^ we believe that the feature reported herein comes from a different type of attached functional group, *i.e.*, a benzoyloxyl radical which can be described as a SWCNT–O–C(=O)–R. This hypothesis is consistent with the literature when considering the stability of BPO-derived oxygen (benzoyloxyl) and carbon (phenyl) radicals, the influence of decarboxylation process and other studies regarding the affinity of oxygen and carbon radicals toward SWCNTs.^31^ An attempt to experimentally corroborate this defect structure is presented in the following part of the paper.

The increase in the 
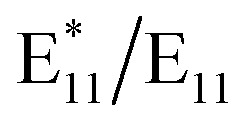
 intensity ratio corresponds to the elevated density of luminescent sp^3^ defects created on the SWCNTs' walls.^54,55^ When a high temperature and substantial BPO concentration (*i.e.*, 100 °C, 15 mM) were used, the high density of defects and probably radical recombination processes affected the course of functionalization, which was observed as the appearance of additional peaks in the PL map (Fig. 1a).^56^ Considering that, as indicated above, the cleavage of the O–O bond in BPO can be altered with both internal (solvent) and external factors (temperature), we decided to capitalize on these relationships.

**Fig. 2:**
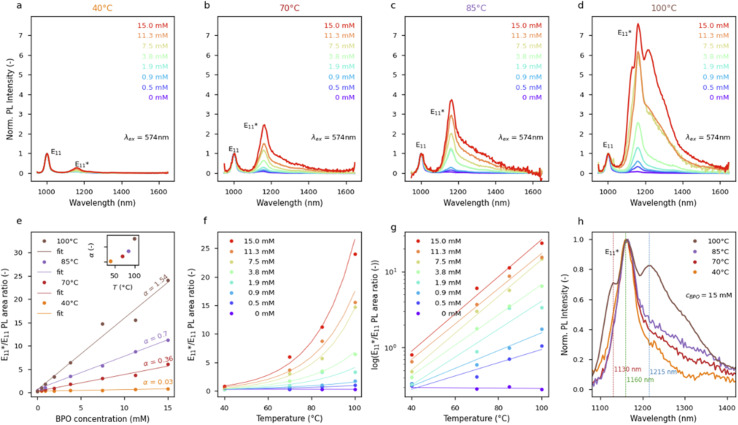
PL characterization of (6,5) SWCNTs functionalized using various concentrations of BPO from 0 to 15.0 mM. Spectra were registered after the reaction conducted at (a) 40, (b) 70, (c) 85, and (d) 100 °C for 1 hour. The ratio of 
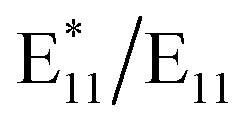
 integrated intensities of light emission as a function of (e) employed BPO concentrations for various temperature levels with linear fits (the inset illustrates the change in the slope *α* of the linear fit function as a function of temperature), (f) temperature for different BPO concentrations with exponential fits. (g) Logarithm of the ratio of 
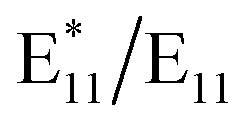
 showing linear correlation with temperature, confirming the autocatalytic nature of the reaction. (h) Comparison of 
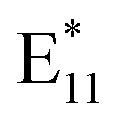
 spectral variability obtained for high BPO concentration of 15.0 mM at various temperatures.

Zaumseil *et al.* showed that the PL intensity ratio can be used as a simple measure of the extent of SWCNT functionalization when comparing data created using a known and fixed excitation power.^20^ Hence, we used it for analysis of the PL spectra of the samples measured using the same parameters (details in the Experimental section). Ideally, for practical application of SWCNTs (*e.g.*, in telecommunication), the spectrum should consist of just one narrow peak, meaning that only one type of structural motif is attached to the surface of monochiral SWCNTs at one preferred configuration (L*^−^, L**, or L*).^9^ Very limited intensities of emerged 
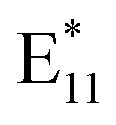
 were observed when the reaction was conducted at 40 °C, which translated into slow radical generation (Fig. 2a). Presumably, for such a low temperature, in order to overcome the kinetic limitations, a higher concentration of BPO and/or a medium capable of better supporting the solvent-induced decomposition of BPO should be used. The possibility of a more facile induction of BPO decomposition in a different solvent environment is verified later in the text.

On the other hand, increasing the temperature to 70, 85, or 100 °C facilitated the materialization of the 
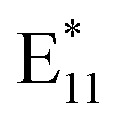
 feature in just 1 hour (Fig. 2b–d). The correlation of the integrated 
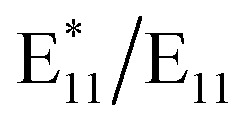
 PL intensity ratio to BPO concentration was linear for all the examined temperature levels (Fig. 2e), which enabled straightforward and precise selection of the desired defect density. In the temperature range from 40 to 70 °C, the slope of the curve changed slightly (especially in the case of low or moderate BPO concentrations), suggesting that many of the radicals formed can still be successively attached to SWCNTs without their excessive accumulation, which could lead to radical recombination (Fig. 2e). An increase in temperature from 85 to 100 °C resulted in a much steeper curve (especially for higher radical concentrations), as defect formation accelerates when both decomposition pathways (unimolecular and bimolecular) occur simultaneously. However, once the temperature exceeded 70 °C (*i.e.*, the SADT value of BPO), for example when the temperature was increased from 70 °C to 85 °C or from 85 °C to 100 °C, the slopes of the linear fit rose significantly (see inset in Fig. 2e). As the number of radicals grew, defect formation accelerated due to the simultaneous occurrence of both unimolecular and bimolecular decomposition pathways. This was associated with the potential for radical recombination or radical transfer, which introduced new radical structures into the reaction system. Raman spectroscopy revealed that 1.9 mM and 7.5 mM BPO treatments at 100 °C produced 76 and 100 functional groups on the SWCNT surface per μm, respectively (Fig. S15†). This estimation is based on the published relationship between the *I*_D_/*I*_G_ ratio, indicative of the level disorder in SWCNTs, and a corresponding defect density.^40^^,^^57^

Moreover, it is noteworthy that the 
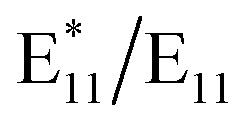
 PL increased in an exponential fashion with the increase in temperature (Fig. 2f), with a significant increase in defect density noted at 100 °C for BPO concentrations above 3.8 mM. The exponential increase with respect to temperature was confirmed by depicting the 
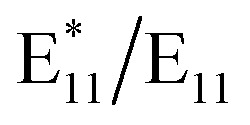
 ratio as a logarithm (Fig. 2g). The observed BPO concentration threshold of 3.8 mM is not an absolute level, but rather indicates the point at which the molar ratio of BPO to SWCNTs exceeds the ability of SWCNTs to effectively scavenge the generated radicals under the standard reaction conditions. To further investigate this phenomenon, we performed additional experiments by varying the concentration of SWCNTs while different initial BPO molar ratios (ranging from 0 to 250) were used for SWCNT functionalization. PL spectra of SWCNTs at low (0.36 μg mL^−1^) and high (0.72 μg mL^−1^) concentrations (Fig. S16†) revealed that higher concentrations of SWCNTs proportionally decreased the extent of SWCNT functionalization. At the same time, the spectral shape was maintained to a large extent.

Determining the kinetics of BPO decomposition in a system containing SWCNTs is complex, as the process is highly dependent on the reaction system's composition, including the concentrations of the initiator and SWCNTs, solvent type, and the presence of compounds, which may interfere with the reaction course. Specifically, in the solid state, BPO undergoes an autocatalytic decomposition, whereas in toluene, its decomposition follows *n*-th order kinetics.^45^ The aspect of BPO decay kinetics of BPO in the absence of SWCNTs is elaborated in the ESI using ^1^H NMR (Fig. S4–S8, Table S1†). The presence of SWCNTs introduces an additional dimension to this reaction that has yet to be characterized from a kinetic perspective. Unfortunately, actual literature does not provide a detailed explanation of how SWCNTs may alter the kinetics of BPO decomposition, although it is suggested that SWCNTs can catalyze decay of BPO.^32,58^ Last but not least, one should consider the presence of residual metallic catalyst particles in the SWCNT materials, which may facilitate this process to an even greater extent. We hypothesize that, in these conditions, the spontaneous and induced decay pathways interfere with each other, and radical formation becomes barely controllable, rendering the reaction outcome more unpredictable. Moderate control of radical reactions caused by high-temperature thermal decomposition of BPO in toluene^44^ was also documented by Shen, who observed repeated deviations between successive runs. In our case, this variability in the obtained PL spectra, even among identically prepared samples, was noted, especially when the reaction temperature significantly exceeded the SADT of BPO (Fig. S17b†).

Furthermore, when a temperature of 100 °C and a BPO concentration above 3.8 mM were used (resulting in an exponential temperature dependence), the emission spectra revealed several new characteristic peaks (especially in Fig. 2d, red line). This provided direct confirmation of the change of course of the radical generation process at temperatures significantly above the SADT of BPO. The resulting high concentration of radicals at high temperatures enabled the formation and attachment of typically less energetically favored defects originating from recombinant (secondary) radicals, leading to a modification of the PL characteristics. In addition, such conditions promoted bimolecular functionalization, resulting in the attachment of a second functional group in the vicinity of the first one, giving rise to the emergence of the 
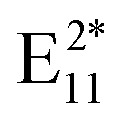
 feature.^9,14^ A relatively long shoulder to the defect-induced PL peak was also present. Berger *et al.* observed a similar “additional red tail that stretches to 1450 nm” at a high concentration of the reactant when creating fluorescence defects using diazonium salts in organic solvents.^20^ Moreover, the emission wavelength of a functionalized material strongly depends on the molecular structure of the attached functional group^6^ and on the binding configuration of the defects in the sp^2^-hybridized SWCNT lattice.^14,34^ This dependence was very well illustrated for the extensively studied aryldiazonium functionalization; when the functional group was attached to the SWCNT wall, creating an sp^3^ defect, another carbon atom in the *ortho* or *para* position to the first one must be saturated with a pairing group, such as a hydrogen atom, an OH group (for the process conducted in water), or another alkyl/aryl group.^14,59^ Density theory calculations showed that each binding configuration leads to a different defect energy, which translates to dissimilar wavelengths of emission.^34^ For example, the *ortho*-L_90_ configuration produces an 
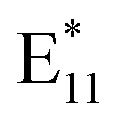
 peak at *ca.* 1160 nm, while *ortho*-L_30_ gives 
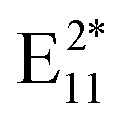
 at *ca.* 1260 nm.^11,27,28^ Most methods of SWCNT functionalization produce a mixture of these configurations.

These results imply that the application of considerable BPO concentration at high temperatures leads to SWCNTs grafted with relatively densely packed functional groups in various configurations, explaining the heterogeneous nature of the recorded PL spectrum (Fig. 2h). Regardless of the concentration and process temperature, we observed the main signal from 
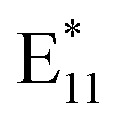
 emission at 1158 nm, which is consistent with our previous observations.^24^ As the process temperature increased, both the kinetic energy of SWCNTs and the concentration of formed radicals increased, which most likely translated into an amplified probability of the formation of *ortho*-L_30_ defects^9^ or defect implantation in close proximity to existing functional groups, which is manifested as a peak positioned at 1215 nm along with clearly marked red tails. The last signal, *i.e.*, at 1130 nm, observed solely for the reaction conducted at 100 °C, most likely arose from the grafting of SWCNTs with C-type radicals (phenyl or benzyl type) formed by radical transfer to the solvent molecule (toluene), or by benzoyloxy radical decarboxylation, according to the previously shown mechanisms (Fig. 1b). The complex character of the BPO decomposition, involving many possible routes, can be evidenced by the multiple isolable side products registered in classical reactions devoid of SWCNTs (Fig. S4–S7†).^43,45,57^

Distinct differences were also noted when SWCNT functionalization was studied as a function of time (Fig. S17†). As expected, the luminescent defects evolve much faster for higher BPO concentrations. In addition to the change in the kinetics of the process, there is a noticeable alteration in the optical properties of the functionalized SWCNTs at high BPO concentrations due to the emergence of a broader spectrum of reactive species above the SADT of BPO.

### 3.1 Influence of moisture and oxygen on the radical formation to produce quantum defects in SWCNTs

This complexity of the reaction system arose from the influence of solvent molecules, which, as previously highlighted, may interfere with the BPO decomposition, while being simultaneously capable of affecting the stability/reactivity of the formed radicals. In addition, even after eliminating the solvent effect, the radical formation mechanism is also prone to environmental factors, such as the presence of oxygen, moisture, acids/bases, or trace metals, all of which may impact the BPO decomposition's kinetics and thermodynamics.^33,35,60–62^ We conducted a series of additional experiments to better understand the reaction mechanism and identify the process conditions giving sharply defined PL peaks. We started by measuring the moisture content of the prepared samples by Karl Fischer titration to estimate the source of moisture and to assess whether the direct addition of water to the reaction mixture could increase its availability, despite its limited solubility in toluene. The results of these measurements are presented in Table S2.†

Functionalization in organic solvents is commonly considered free from the impact of aggressive hydroxyl radicals (˙OH) due to the absence of water. However, BPO is typically stabilized with water (here 25%) to minimize the risk of explosion during long-term storage. Therefore, a considerable amount of moisture was present in the reaction mixture. Additionally, moisture from the air can be adsorbed by SWCNTs or solvents during storage/sample preparation (Table S2†). The solubility of water in toluene increases with temperature, which may also explain the appearance of hydroxyl radicals when SWCNTs are treated at high temperature.^63^

To remove water from BPO, it can be dissolved in a neutral solvent, such as chloroform or dichloromethane, followed by the addition of anhydrous magnesium or sodium sulfate until clumping ceases. The solution can then be filtered and evaporated at a low temperature (below 40 °C). Alternatively, BPO can be synthesized independently following established procedures from the literature.^64,65^ However, several key principles for handling peroxides should be observed, as extensively outlined in the literature.^66^ In particular, it is advised to work with small quantities (up to 1 g), avoid incompatible substances (bases, acids, oxidizers, heavy metals), mitigate exposure to decomposition-inducing conditions such as light and heat, and store BPO in appropriately sized plastic containers under controlled conditions.

**Fig. 3:**
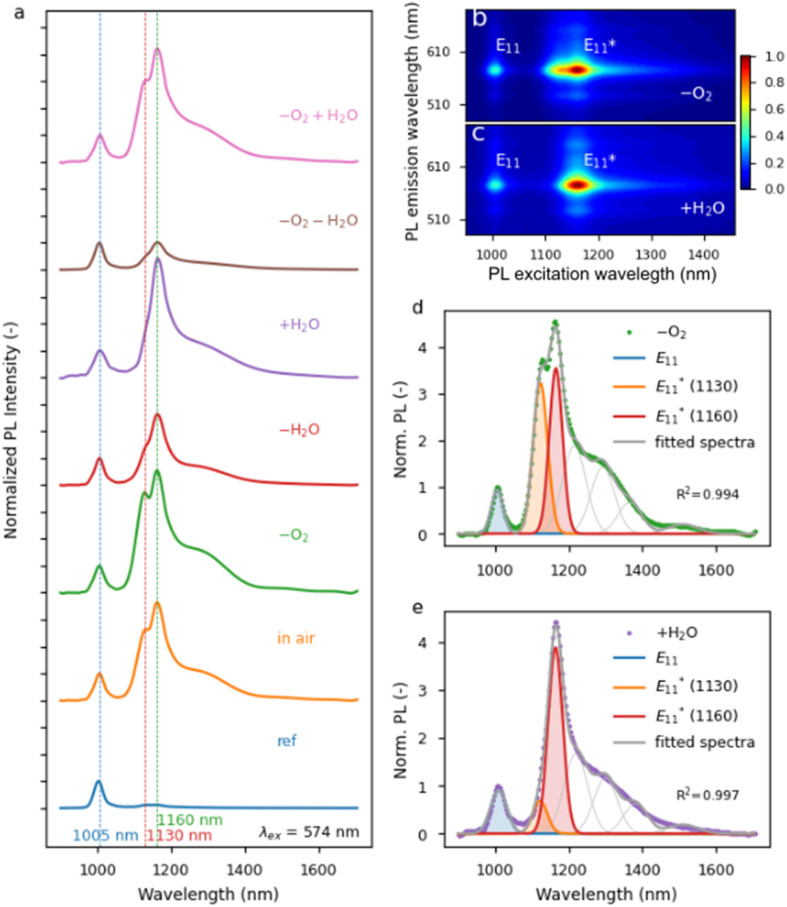
(a) Normalized PL spectra of (6,5) SWCNTs functionalized with 3.8 mM BPO at 100 °C under various conditions. The wavelengths at which the main peaks were found (E_11_ – 1005 nm, 
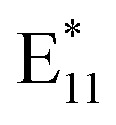
 – 1130 nm and 1160 nm) are marked with dashed lines. PL excitation–emission maps of the samples prepared with (b) argon and (c) addition of water. PL spectra of the samples prepared with (d) argon and (e) addition of water fitted with component peaks: E_11_, 
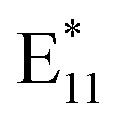
 consisting of two peaks at 1130 and 1160 nm, and 
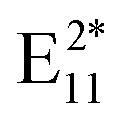
 peaks at 1285, 1330, and 1355 nm.

The ˙OH created in this manner can attack SWCNTs and cause unintentional functionalization, leading to different addend species on the SWCNT surface, which can make the PL spectra more diverse. Moreover, hydroxyl radicals can react with BPO or with its decomposition by-products (benzoic acid, benzoate esters, or biphenyl) and generate secondary chemical compounds, such as phenols, thereby affecting SWCNT properties and possibly the functionalization process.^67^ This is an essential aspect to consider since hydroxyl radicals generated from moisture can promote the scission of BPO, increasing the abundance of radical species.^52,57^ On the other hand, the effect of oxygen on BPO decay has been studied more thoroughly, and, in most cases, it has been identified as an inhibitor of radical pathways.^68,69^ Hence, in theory, it should hamper the BPO decomposition process, and, consequently, hinder the capacity for SWCNT functionalization. However, some reactants and solvents are prone to the presence of oxygen, and a wide range of different reactive species can be created in contact with oxygen, *e.g.*, benzylperoxyl radicals.^70^ To evaluate these effects in the context of SWCNT functionalization for the first time, we conducted the thermal functionalization at 70 and 100 °C using 3.8 mM BPO under various conditions (Fig. 3 and S19,† respectively), *i.e.*, the SWCNT grafting was carried out in the absence of oxygen (“–O_2_”, flushed with argon), in the absence of water (“–H_2_O”, dried with molecular sieves) or with the intentional addition of H_2_O (“+H_2_O”). More details can be found in the Experimental section.

The methodology of sample preparation had a major impact on the process and the density of defects obtained at 70 °C after 1 h of the reaction, as shown in Fig. S19a.† This can be clearly seen given that the 
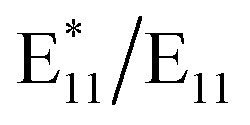
 ratio varied over a large range, *i.e.*, from 0.4 to 1.0. The 
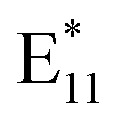
 peak at *ca.* 1160 nm dominated all the PL spectra presented in Fig. S3,† but other spectral features of low intensity at longer wavelengths (1200–1450 nm) could be distinguished. However, when the spectra were normalized, in most cases, they overlapped over almost the entire range (Fig. S19b and d†), which can be attributed to the controlled radical generation and, most likely, the marginalization of induced bimolecular BPO decomposition, which could generate secondary radicals, thereby making the spectrum more heterogeneous. The sole differences were seen on the right slope of the main peak from functionalization, which originated from the formation of new peaks with maxima of around 1285 nm. Interestingly, these features were least abundant for both the samples with the addition of water, even though functionalization proceeded most rapidly under such conditions. This finding may be related to the role of water as an effective hydrogen atom source for the saturation of the radical formed on the surface of SWCNTs after attachment of the aryl group.^61,69,71–73^ This hypothesis was further confirmed by the PL spectrum of the sample functionalized in the absence of H_2_O, where the opposite effect was noted. Compared to the sample prepared in air, after oxygen removal, more benzoyloxy radicals were available to bond with SWCNTs, which was manifested by a slightly higher 
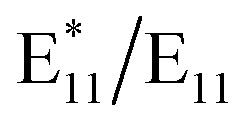
 intensity ratio. However, this effect was subtle. When mixed strategies were implemented, the results were consistent. By reducing the amount of oxygen and removing water, those two effects balanced each other out. Since oxygen acted as a radical scavenger,^43^ the extent of functionalization by BPO increased under inert conditions, but simultaneously, the reduction of water content by the application of molecular sieves tamed the influence of the ˙OH radical. To further verify the influence of water, we synthesized BPO with a low moisture content. The synthesis procedure, along with structural characterization and water content analysis, is detailed in the ESI.† In this case, we also observed notable differences in the optical characteristics of the functionalized at 100 °C (6,5) SWCNTs (Fig. S20†).

Higher 
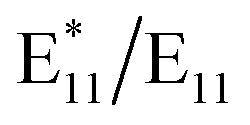
 intensity ratios and larger spectral diversity were observed when the SWCNTs were modified at an elevated temperature of 100 °C (Fig. 3a, S3c and d†). Under ambient conditions (“in air”), the PL spectra contained a complex feature in the 
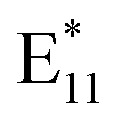
 region composed of two distinctive peaks at *ca.* 1130 and 1160 nm. Furthermore, a tail containing probably three peaks extending in the 1200–1400 nm range was present. Moreover, in the absence of oxygen, peaks from all the above mentioned defects were also developed, with the highest relative intensity of both 
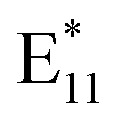
 components among the evaluated samples. This finding was consistent with previous results, meaning that oxygen retarded the decomposition of BPO, but its influence was subtle in the organic environment.^35,43^ Additionally, because the spectral line shape was similar to the one obtained in air, we concluded that at high temperatures the oxygen does not significantly impede the reaction of SWCNTs with BPO. This may be due to the fact that the concentration of radicals produced under these conditions is greater (
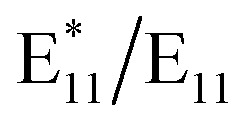
 ratio is much higher) since the solubility of gases in liquids decreases with temperature. Replacing the oxygen with argon seemed to increase the proportion of the peak at 1130 nm (Fig. S3c†), which was in line with the literature data reporting that carbon-based radicals are rapidly trapped by molecular oxygen or other reactive oxygen species.^74^ This trend was particularly evident when comparing samples with the addition of water with the ones processed under inert conditions. Again, when water was eliminated from the system, the emergence of peaks originating from defects was reduced, as expected. In contrast, when water was intentionally added to the mixture, the appearance of 
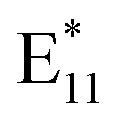
 at 1160 nm was boosted, which corroborated the results obtained at a lower temperature. Interestingly, the peak at 1130 nm was not prominent in either case, which made the functionalization reaction more selective. This phenomenon was observed in PL maps (Fig. 3b – argon and Fig. 3c – intentional water addition), where a differently shaped 
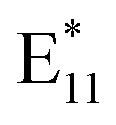
 peak was noted. Fitting PL spectra extracted from these maps allowed for the visualization of these peaks' contribution to PL emission (Fig. 3d and e).

The 
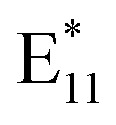
 component at 1130 nm appeared after modifying the SWCNTs in air and upon reducing the amount of oxygen (“–O_2_” and “–O_2_ + H_2_O”), so these conditions at 100 °C appeared to favor the formation of a different defect configuration. In explaining the origin of the peak with a maximum at 1130 nm, it is helpful to consider the position of the peaks formed when the functionalization is performed with different alkyl or benzyl groups (*e.g.*, bromobenzene or α,α′-dibromo-*o*-xylene).^23,75^ Most likely, this defect originates from the functionalization of SWCNTs with a benzyl radical, which, at low temperatures, *i.e.*, 70 or 85 °C, is insufficiently reactive due to stabilization with several resonance structures. Furthermore, at these temperatures carbon-based radicals are probably present in too low concentration to effectively attack the surface of the SWCNTs (Fig. 2 and S3†). On the other hand, this does not prevent the participation of benzyl radicals in the induced decomposition of BPO,^76^ as shown before (Fig. 1b). At 100 °C, the activity and concentration of benzyl radicals became sufficient to directly functionalize the SWCNT surface and produce a new type of defect (*via* C-type linker). However, given the susceptibility of this benzyl radical to oxidation,^70^ the appearance of defects originating from the benzyl group depends largely on the presence of oxygen and water in the reaction mixture.

### 3.2 Influence of mixed-solvent environment on the radical formation to produce quantum defects in SWCNTs

The role of the solvent in the process of modification of the PL emission from SWCNT is multidimensional. Firstly, it was shown that the solvents can take part in the reaction of functionalization by providing the pairing group for covalent modification, *e.g.*, a hydrogen atom lost by the solvent molecule can saturate the open-shell system created upon binding the “main” functional group. Consequently, the 
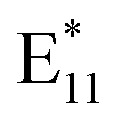
 and 
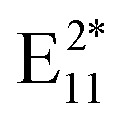
 peak positions are influenced by the chemical nature of the attached groups and their binding configurations.^14,59^ Secondly, the optical properties of SWCNTs are sensitive to the microenvironment surrounding them. Solvent properties, such as polarity, strongly impact the conformation of the wrapping polymer, thereby affecting the optical properties of SWCNTs^38^ and possibly also the reactivity of the polymer@SWCNT system. Solvent polarization influence was also observed in aqueous dispersions of SWCNTs.^18^ Moreover, solvent molecules are also capable of affecting the defect-state relaxation dynamics.^77^ Additionally, we must consider the compatibility of the solvents with the polymer used to disperse SWCNTs. Polar or very non-polar solvents usually cause polymer precipitation through self-aggregation, making the SWCNT suspension unstable, which impacts the PL spectra or prevents the possibility of PL measurement altogether.

An aspect that has not been extensively discussed in the literature is the effect of wrapping SWCNTs with conjugated polymers on the resulting morphology and types of defects. To eliminate the possibility of interactions between the polymer and the BPO-generated radicals, we performed functionalization of the crude material in the absence of the polymer. This was followed by washing away the radical source and redispersing SWCNTs with PFO-BPy for characterization. The functionalization results normalized to the E_11_ and 
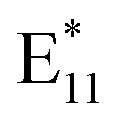
 peaks (Fig. S21†) showed that the presence of PFO-BPy did not affect the course of SWCNT functionalization with BPO, as the spectra had analogous shapes.

In the experiments described so far in this study, BPO was dissolved in toluene, in which SWCNT dispersions facilitated by the deposition of polymer molecules were also prepared. To investigate the possible impact of the liquid medium on the extent and type of functionalization, BPO was dissolved in various organic solvents (called co-solvents in the remainder of this paper), combined with SWCNT in toluene (the co-solvent : toluene vol. ratio was equal to 1 : 1), and the mixture was subjected to a 100 °C treatment for an hour. The properties of the employed co-solvents can be found in Table S3.† Each mixed-solvent system resulted in different PL emission spectra, but the optical properties of the solvent themselves did not seem to affect the data obtained (Fig. S22 and S23†). We started by sorting the results according to the dielectric constants of the co-solvents (Fig. S4†). For hexane, diphenyl ether, cyclohexane, and decalin, co-solvents with low dielectric constants in the range of 1.89–2.23, similar spectral shapes were obtained, with a dominating 
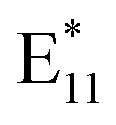
 feature positioned at 1160 nm accompanied with an extensive tail up to about 1450 nm. Furthermore, for pure toluene (*ε* = 2.38), as well as toluene mixed with *o*-xylene (*ε* = 2.57) and tetralin (*ε* = 2.77), similar spectra to those observed in toluene were obtained, with comparably high intensities of 
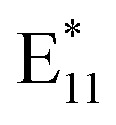
 features in the case of toluene and *o*-xylene mixture. A wide spectral variety and low relative intensities of defect peaks were obtained with tetrahydrofuran (THF, *ε* = 7.40) addition. For even larger values of dielectric constant, dichlorobenzene (DCB, *ε* = 9.93) and acetonitrile (*ε* = 37.5), a large spectral variety was noted, and significantly lower relative values of the 
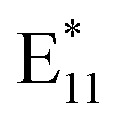
 PL intensity were obtained using these co-solvents. It seems that in relatively polar solvents (here, DCB or acetonitrile), a variety of moieties in different configurations were attached to the SWCNT walls, resulting in a high defect density. In summary, although the solvent dielectric constant matters with regard to the result of PL modification, it does not seem to play the leading role due to the obvious spectral inconsistencies.

At this point, we decided to take a closer look into the effect of the solvent molecular structure. Solvents have a key impact on the behavior of radical initiators such as BPO. A good insight into this issue is provided by the work of Bartlet and Nozaki,^35,43^ who examined the influence of solvents on the induced decomposition of BPO. They discovered that the benzoyloxy radicals could attack the solvent molecules, leading to the creation of various radical species. On the other hand, phenyl or solvent-based radicals can also attack BPO molecules to form benzoyloxy radicals and an unreactive residue, *i.e.*, phenyl benzoate or benzyl benzoate.^33^ The rate of induction of BPO decomposition decreases in the following order: amines > ethers, alcohols, phenols > most aliphatics > most aromatics > halogenated solvents. Thus, most alcohols and amines react violently and exothermally with peroxides, which makes them incompatible as solvents for SWCNT functionalization. In the context of ethers, the situation is more complex, and their structure must be considered directly. The aliphatic ones show markedly different behavior from the aromatic ones, and the pivotal factor is the presence of alpha hydrogen atoms.^43^ The structure of the ether molecule affects the propensity of BPO decomposition. Diphenyl ether or anisole experiences about 15% decomposition at 80 °C after 60 minutes, whereas this value can be as high as 82% for reactive dioxane. At the same time, remarkably high values of the degree of decomposition are reached for aliphatic ethers, *e.g.*, diethyl ether was already decomposed after about 10–15 minutes.^43^

It is also important to mention a few points discussed in the abovementioned articles that are relevant to the interpretation of our results. Firstly, the presence of radicals accelerates the decomposition of the peroxide molecule, so it is important to determine how fast the formed radicals will be consumed by the SWCNTs. In addition, if the reactivity of the radical toward SWCNT functionalization is not sufficient, then, in the case of non-reactive solvents, the recombination of two benzoyloxy radicals recreating the initiator molecule has considerable implications in terms of the duration and efficiency of its breakdown process. For a more detailed and quantified discussion of how each of the solvents we tested induces BPO decomposition, one may refer to the literature further exploring various aspects of this topic that are outside of the scope of this article.^35,43^

**Fig. 4:**
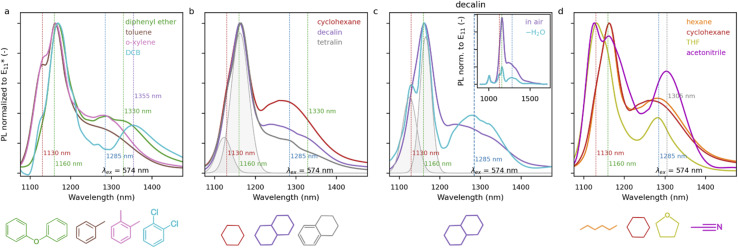
Comparison of defect emission spectra of (6,5) SWCNT functionalized with 3.8 mM BPO in mixed-solvent environments with chemical structure of the solvents used to dissolve BPO (co-solvents with toluene), shown below. Spectra are compared for (a) diphenyl ether, toluene, *o*-xylene, and DCB, (b) cyclohexane, decalin, and tetralin and (d) hexane, cyclohexane, THF, and acetonitrile co-solvents. (c) The spectra obtained in the additional experiment with decalin as a co-solvent: in air and dried (“–H_2_O”). The peaks at 1130 and 1160 nm are fitted to both spectra obtained in decalin (in b and c) for their relative height comparison. Dashed lines show the positions of the discussed peaks.

A small degree of BPO decay, *i.e.*, in the range of 13–18% after 1 h of heating at 80 °C, was found for halogenated aliphatic and aromatic hydrocarbons, such as chloroform or chlorobenzene, but, interestingly, similar results were also obtained in benzene or toluene.^35^ Comparison of the obtained defect densities in toluene, or toluene mixed with *o*-xylene, tetralin, and diphenyl ether (measured by the 
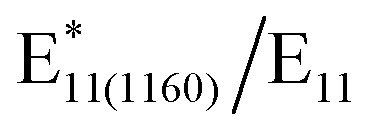
 ratio, Fig. S4†) showed that the results correlated with BPO decomposition reaction rates at 100 °C. Despite the similar decomposition rate among those solvents, only toluene and toluene : *o*-xylene additionally promoted the emergence of the peak at 1130 nm (Fig. 4a). We anticipate that the methyl groups present in these solvents are crucial for carbon-based radical (C-radical) formation (in the absence of oxygen) *via* the chain transfer reaction.^78^ During the radical functionalization of the SWCNT surface, the breakdown of the double bond releases an unpaired electron that appears near the defect and must abstract a hydrogen atom from another molecule. It is likely that a hydrogen atom from the methyl groups in the solvent molecule may participate in this transfer. This causes two results: firstly, the uptake of H˙ to saturate the defect (monofunctionalization), secondly the generation of a benzyl radical, which can either participate in subsequent functionalization with the formation of a peak at 1130 nm or facilitate BPO decomposition. When cyclohexane and decalin, which contain aliphatic rings, were used as co-solvents, the obtained high ratios of the 
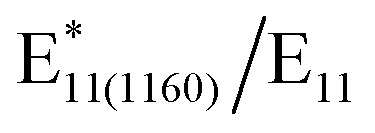
 PL intensity were in line with the high reaction rates of the solvent-induced BPO decomposition highlighted previously. In contrast, this effect was somehow negated in toluene : tetralin mixture due to the presence of the aromatic ring (Fig. 4b). For toluene : decalin, the 
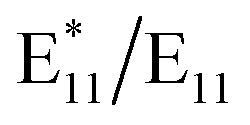
 peak area ratio was the highest among the tested reaction media. This may indicate that it induces the BPO breakdown, while the decalin radical itself is not reactive enough or too bulky to attach SWCNTs.

To better understand the nature of decalin, BPO dissolved in this solvent stored over molecular sieves was employed for the functionalization. The aim was to probe the role of water in the creation of benzoyloxy radicals. Similarly, as in toluene (Fig. 3a), the removal of water from the reaction caused limited emergence of the 
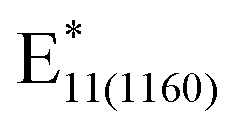
 (Fig. 4c, inset). The peaks fitted at *ca.* 1130 and 1160 nm for decalin are shown in Fig. 4b and c, for standard (“in air”) and dried (“–H_2_O”), respectively, for comparison of these peaks' relative intensities.

Furthermore, the tail extending in the 1200–1450 nm range, most visible in the case of using cyclohexane as the co-solvent (Fig. 4b), probably originated from the high concentration of benzoyloxy radicals in this solvent, which increased the chance of bimolecular functionalization, *i.e.*, the formation 
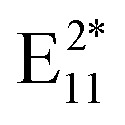
 defects. They can be generated by attachment to the surface of two benzoyloxy radicals or a benzoyloxy radical and solvent radical. For the latter moiety, its effective participation in the SWCNT grafting process hinges upon it exhibiting a sufficiently compact or flexible structure. This factor becomes clear after comparing the practically identical shape of the PL spectra after SWCNT functionalization for hexane and cyclohexane (Fig. 4d) with those from decalin (Fig. 4b).

The last of the co-solvents that generated very interesting functionalization spectra were THF and acetonitrile (Fig. 4d). While the application of the former translated into a modest defect density in functionalized SWCNTs, acetonitrile was one of the most effective co-solvents after decalin, cyclohexane, and hexane (Fig. S21†). The uniqueness of these solvents is also related to the dominance of the peak at 1130 nm instead of the usual prevalent one at 1160 nm. In the case of acetonitrile, we also observed the highest peak contribution of 
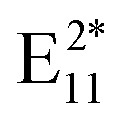
 out of all the investigated solvents. Although this particular solvent has not been considered in previously mentioned studies by Bartlet, Nozaki, and Swain,^33,35,43^ the latter analyzed the decomposition process of BPO in dioxane in the presence of benzyl cyanide (sharing somewhat similar functional groups). This medium was identified as a quite effective inhibitor of the solvent-induced decomposition process. Interestingly, valuable insights to elucidate this aspect can be found in a paper that examined the BPO-initiated polymerization of methyl methacrylate in acetonitrile.^79^ The authors observed that acetonitrile underwent a chemical reaction in the presence of BPO, and that the by-products formed accelerated the process of further decomposition of this radical source. Thus, the high reactivity of acetonitrile in the context of initiating the radical formation process most likely, together with this solvent radical having the smallest size among all those formed, results in favoring the attachment of alternative radical structures to the surface of the SWCNTs.

**Fig. 5:**
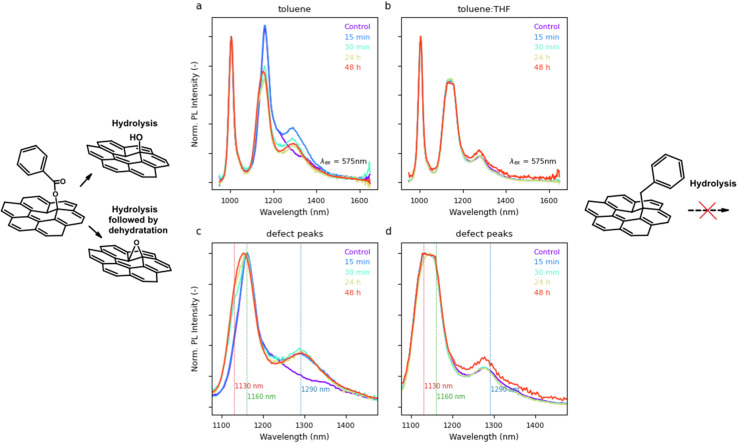
The PL spectra of (6,5)-functionalized SWCNTs after hydrolysis mediated by TMAH solution in methanol. Hydrolysis was carried out on two samples with different mechanisms of functionalization: (a) occurring *via* a benzoyloxy radical in toluene (dominant peak at 1160 nm) or (b) by a solvent-derived radical (dominant peak at 1130 nm) in a toluene : THF mixture. In the latter case, we hypothesize functionalization by C–C bond formation, which is resistant to hydrolysis. The corresponding spectra normalized to 
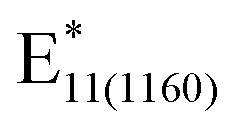
 peak intensity are shown in panels (c) and (d) for shape comparison.

To further confirm that the peak emerged at 1130 nm, observed upon functionalization with BPO at 100 °C, was the effect of the SWCNTs' functionalization with benzyl groups, we conducted the reaction with BPO in pure benzene. Benzene has no methyl group, so we expected it to be unable to generate the peak at 1130 nm, even with a high degree of functionalization. The SWCNTs were transferred to the desired solvent by the evaporation of toluene and redispersion. BPO was dissolved in benzene, mixed with SWCNTs, and the reaction was conducted without further modifications of its protocol. The reactivity of the BPO/SWCNT system in benzene was significantly higher than in toluene (Fig. S5†). Since this solvent is not particularly effective in the induced decomposition of BPO,^35^ we believed that the effective concentration of BPO with respect to SWCNT concentration was higher than in toluene. SWCNTs tended to agglomerate in benzene after some time, indicating that it had poor capacity to solvate SWCNTs. After 1 hour of the reaction, no PL signal was observed from functionalized SWCNTs, meaning most of the SWCNTs suffered from excessive functionalization, so the reaction time was reduced to 15 minutes, and the SWCNTs' concentration was raised to about 0.72 μg mL^−1^ (optical density of 0.4 in the case of non-functionalized (6,5) chirality). For reliable comparison, the reaction in toluene was also repeated with a higher SWCNT concentration. Under these conditions, the PL peak at 1130 nm emerged when 15.0 mM BPO was used (Fig. 5a and d), but was not seen for a lower concentration equal to 3.8 mM. This suggests that its appearance requires a high abundance of BPO with respect to the SWCNTs. Possibly, when a small BPO concentration was used, the O-type benzoyloxy radicals (accountable for the 1160 nm peak) preferentially attacked the SWCNT surface.^14^ On the contrary, when a large abundance of BPO species was present, some attacked the toluene molecules, leading to the creation of benzyl radicals, which then competed for the SWCNTs' functionalization, giving rise to the PL peak at 1130 nm. When benzene was used as a solvent for the functionalization reaction, no relative increase of PL intensity at 1130 nm was noted despite the high BPO concentrations and relatively extensive degree of functionalization of the SWCNTs (Fig. S24b and e†). To eliminate the problem of the low dispersibility of SWCNTs by benzene molecules (caused by the marginal solubility of PFO-BPy in it), the same reaction was repeated in chlorobenzene. This solvent appeared to be optimal, as it provided even better solubility of the conjugated polymer than toluene while having no methyl groups. For this solvent, also, no PL emission was noticed at 1130 nm (Fig. S24c and f†), which increased our confidence in the solvent-mediated grafting of SWCNTs producing this unusual feature.

To experimentally verify the structure of the linkage between SWCNTs and the newly attached functional group, we decided to check its susceptibility to hydrolysis analogously to our previous publication.^24^ This time, however, we had access to functionalization that originated from, most likely, benzyl or phenyl radicals (as well as other solvent-specific carbon-based radicals), which prevailed in the case of BPO functionalization carried out in a mixture of toluene and THF. For this purpose, we selected two samples that had definite differences in the position of the main peak from the functionalization, *i.e.*, 1130 nm for the previously discussed functionalization in a toluene : THF mixture and the classical functionalization in toluene with the predominance of the peak at 1160 nm. After adding 4 μL of tetramethylammonium hydroxide (TMAH) in methanol to the reaction mixtures, PL spectra were recorded at various times to see if any spectral change occurred (Fig. 5). Indeed, in the case of the sample functionalized in toluene, we observed a decrease in intensity for the dominant peak at 1160 nm and, in addition, the appearance of a new one at around 1280–1300 nm (Fig. 5a and c). Given its location, this new signal could come from additional modification of SWCNTs in close proximity to the already present defects or due to the formation of an epoxide group, producing a divalent defect.^80,81^ The disappearance of the 
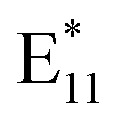
 signal confirmed the labile nature of the functional group, supporting the hypothesis that the 1160 nm peak comes from the benzoyl group attached to SWCNTs *via* a hydrolyzable C–O bond. In contrast, for the sample with a high intensity of the 1130 nm peak, we observed no change in the fluorescence spectrum (Fig. 5b), confirming the presence of a robust C–C bond, likely coming from a benzyl group attached to the surface. To rule out inhibition of the hydrolysis process by THF, we performed an additional experiment for a sample functionalized in toluene, which was diluted with tetrahydrofuran before the addition of TMAH. Hydrolysis was also observed, which strongly suggests that the previously stated hypothesis is correct.

## 4. Conclusions

The results of our research shed new light on the complexity of the functionalization of SWCNTs, which is widely performed in order to enhance their photonic characteristics. This complexity is undoubtedly both an advantage and a disadvantage of the studied BPO-based reaction system because it requires substantial comprehension and intuition to achieve the desired degrees of functionalization (the density of defects, but also functionalization selectivity). On the other hand, the multitude of parameters allows a high degree of customization and optimization of the functionalization process to graft the SWCNTs in the desired way.

The full potential is revealed in the controlled course of functionalization at 70–85 °C, where the degree of functionalization increases linearly with the concentration of the radical source, and the rate of reaction is in the initial period of exponential growth (Fig. 2f). This relationship is attributed to the predominance of controlled unimolecular decomposition of BPO under these conditions, without accumulation of the formed radicals, which translates into high reproducibility of the results obtained. In contrast, functionalization carried out at 100 °C is characterized by a dynamic exponential increase in the number of attached defects and limited reproducibility.

Furthermore, we confirmed that the absence of oxygen and the presence of water is beneficial for the efficiency and selectivity of the functionalization process at moderate reaction temperatures. At higher temperatures, the presence of water was also beneficial, while the removal of oxygen due to its decreased solubility in the liquid medium negatively affected the functionalization selectivity.

Subsequent investigation of the effect of solvent type on the SWCNT functionalization outcome revealed that the peak at 1130 nm comes from functionalization with a C-type radical, *i.e.*, benzyl, which sheds considerable insight into the mechanism of this process. The presence of this defect structure was experimentally verified by hydrolysis studies. However, the role of the solvent is not limited to introducing additional functionalization possibilities. The results of the runs conducted in acetonitrile and THF showed that the solvent can independently influence the prevalent type of generated reactive species, determining the PL characteristics of the obtained materials. Interestingly, when decalin was used as a solvent, it accelerated the breakdown of BPO into radicals, increasing their concentration while not affecting the structure of the groups attached to the surface. Consequently, the choice of solvent is very important for SWCNT functionalization. The obtained results highlight that it may be highly beneficial to consider organic media other than the typically employed toluene to chemically modify SWCNTs to gain even more control over the properties of SWCNTs, directly increasing their application potential.

## Data availability

The data supporting this article have been included as part of the ESI.†

## Author contributions

P. T., A. D., and D. J. conceptualized the study. D. J. supervised the investigation. P. T. and A. D. performed the research. P. T., A. D., and D. J. co-wrote the paper. All authors analyzed the data, discussed the results, and commented on the manuscript.

## Conflicts of interest

There are no conflicts of interest to declare.

## Supplementary Material

SC-016-D4SC04785K-s001
