# Supplementary material for: Unraveling aryl peroxide chemistry to enrich optical properties of single-walled carbon nanotubes
Source: Chem Sci. 2024 Dec 11;16(3):1374–89. doi: 10.1039/d4sc04785k (PMC11653410; doi:10.1039/d4sc04785k)
Supplement: SC-016-D4SC04785K-s001 [file SC-016-D4SC04785K-s001.pdf]

Supplementary Information (SI) for Chemical Science.
This journal is © The Royal Society of Chemistry 2024

(Supporting Information)

Unraveling Aryl Peroxide Chemistry to Enrich Optical Properties of Single-Walled Carbon Nanotubes

Patrycja Taborowska [a,&], Andrzej Dzienia [a,&,*], Dawid Janas [a,*]

a Department of Chemistry, Silesian University of Technology, B. Krzywoustego 4, 44–100, Gliwice, Poland

[&] both authors contributed equally

* Corresponding authors: Andrzej.Dzienia@polsl.pl, Dawid.Janas@polsl.pl

Table of Contents

1. Characterization of self-synthesized PFO-BPy and BPO 2
2. Analysis of BPO decay kinetics 5
3. Analysis of interactions between PFO-BPy6,6' and generated radicals 10
4. Determination of water content via Karl Fischer titration 15
5. Characterization of (6,5) SWCNT dispersion before and after chemical modification 16
6. The influence of the relative amount of (6,5) SWCNT with respect to BPO on the course and extent of SWCNT functionalization at 100°C 17
7. Functionalization of (6,5) SWCNT dispersion with BPO at 70°C and 100°C 18
8. Kinetics of (6,5) SWCNT dispersion functionalization with BPO at 100°C........ 18
9. The influence of processing conditions on the functionalization of (6,5) SWCNTs 19
10. Functionalization of SWCNTs in mixed-solvent media 20
11. Literature........ 24

1. Characterization of self-synthesized PFO-BPy and BPO

The ^{1}H Nuclear Magnetic Resonance (^{1}H NMR) spectra of the polymer (PFO-BPy6,6'), BPO, and its decomposition products were acquired using an Agilent 400-MR spectrometer operating at 400 MHz, with toluene-d_8 as the solvent. The spectrometer was equipped with a OneNMR probe, ProTune automatic probe tuning, and VnmrJ 3 software. Subsequent spectral processing was conducted using the Mestrenova software suite. Chemical shifts were recorded in ppm, referenced to the residual methyl group peak of toluene-d_8, set at 2.09 ppm. Standard experimental conditions (64 scans, T=20°C) were applied.

The ^{1}H NMR spectrum of the PFO-BPy6,6' polymer, synthesized in-house according to the earlier described procedure [1], is presented in Figure S1. The molecular weight of the polymer was assessed by gel permeation chromatography (GPC), yielding a weight-average molecular weight (M_w) of 98.5 kg/mol, a number-average molecular weight (M_n) of 35.0 kg/mol, and a polydispersity index (Đ) of 2.82.

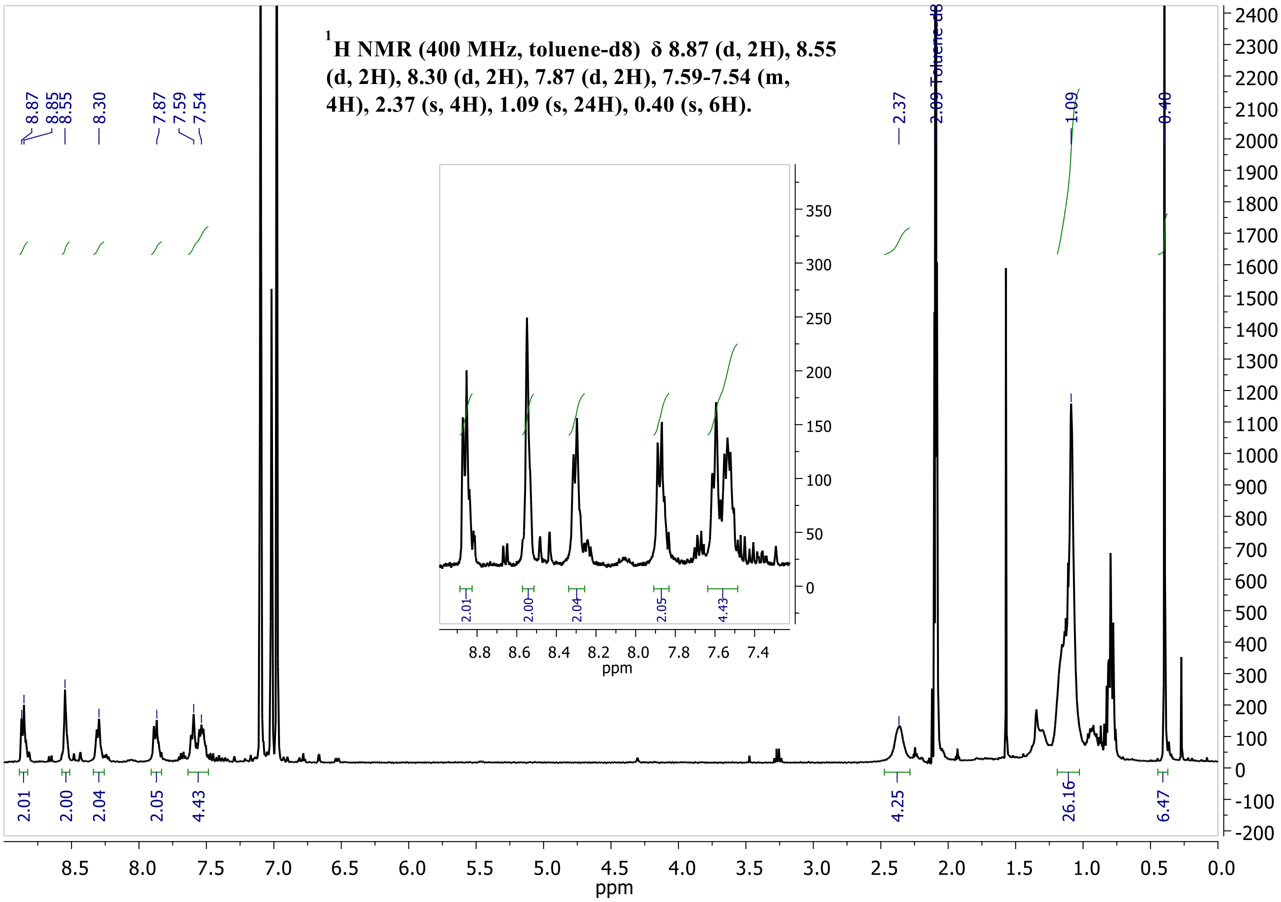

Figure S1 ^{1}H NMR spectrum of PFO-BPy6,6' synthesized in-house.

The ^{1}H NMR spectra of the BPO synthesized according to the reported method [2,3] are presented in Figures S2 and S3. Spectra of unheated BPO were registered in toluene d_8, and signals to individual protons were assigned (Figures S2-S3).

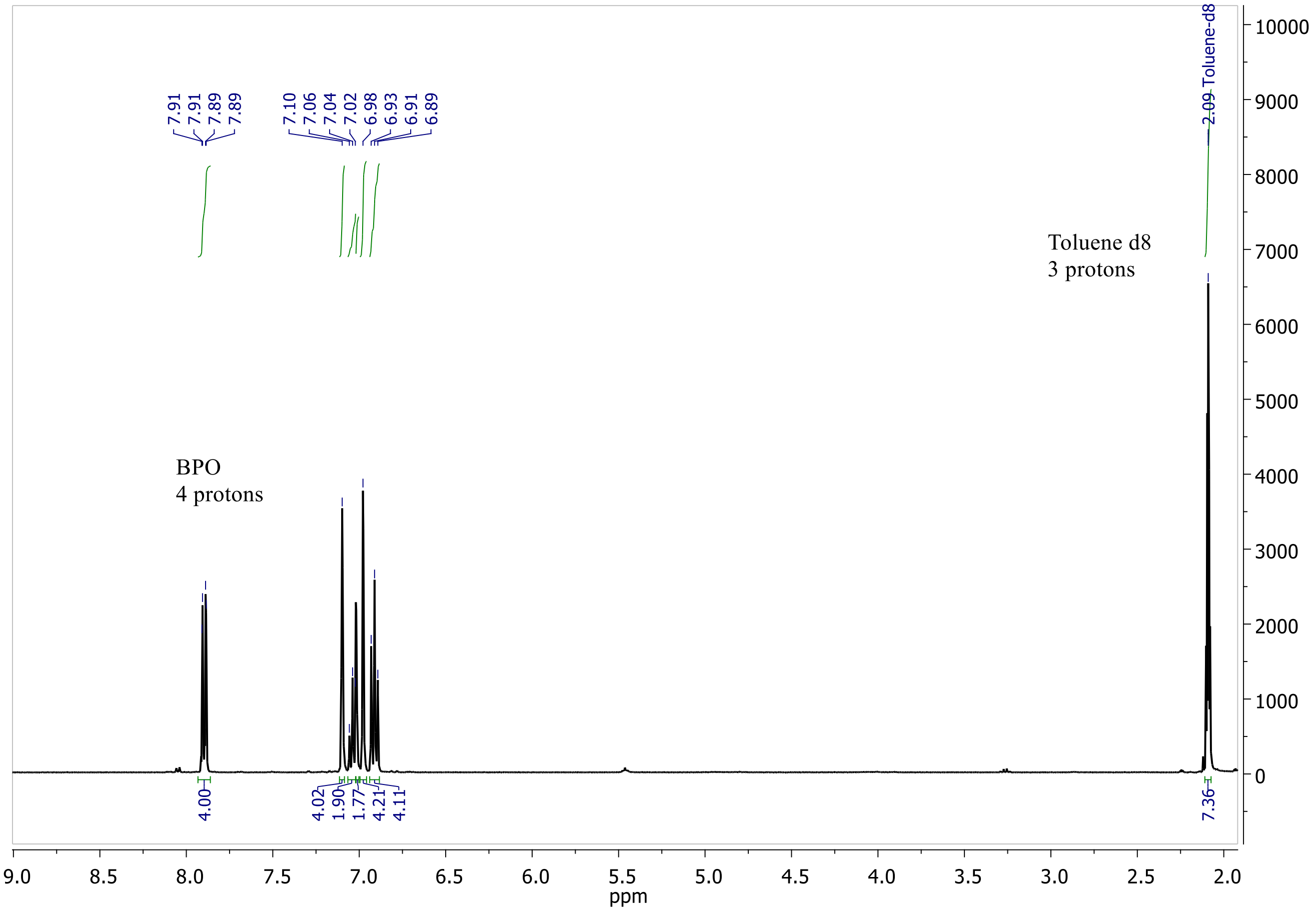

Figure S2 Full ^{1}H NMR spectrum of BPO dissolved in toluene-d_8.

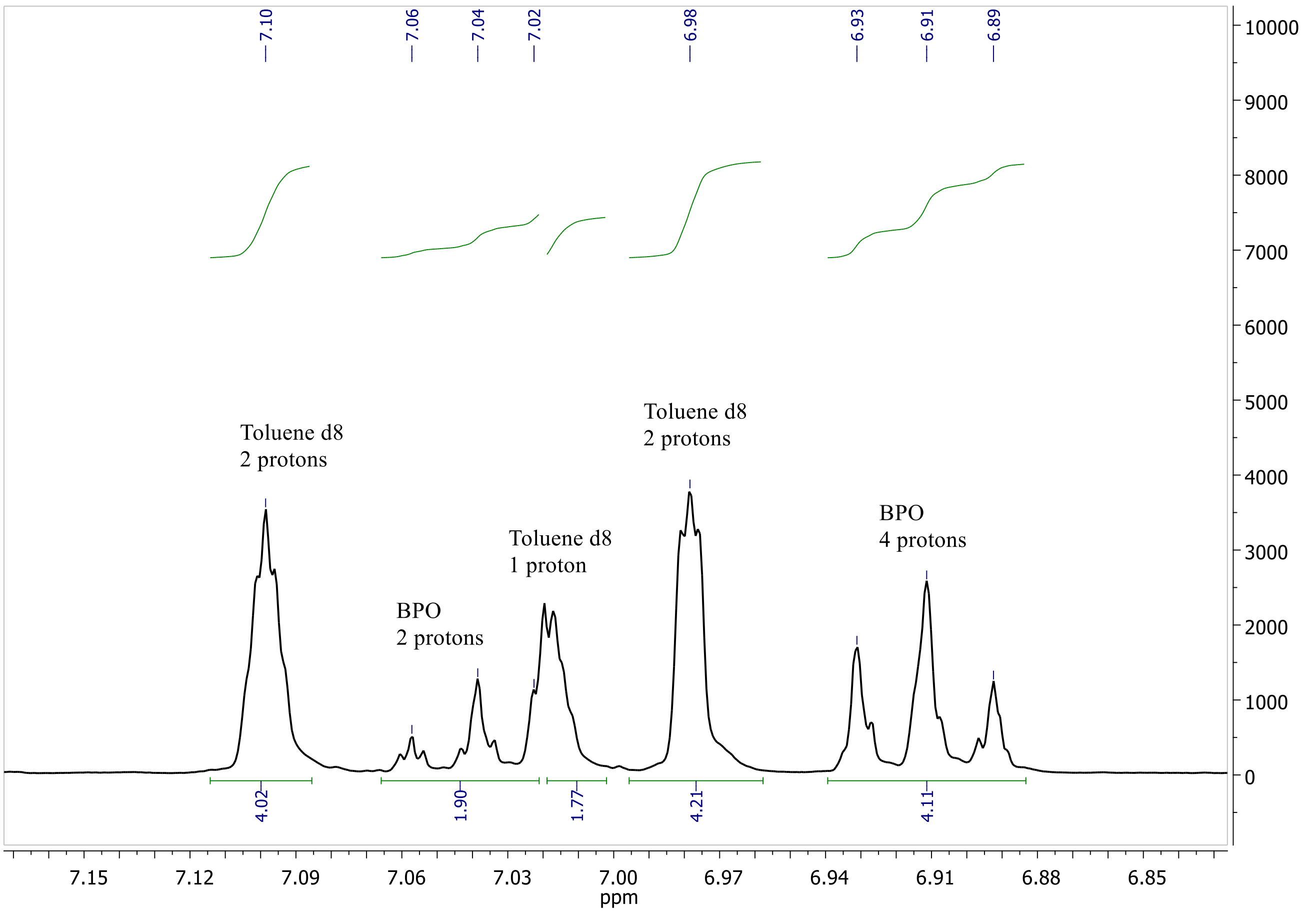

Figure S3 Magnification of the ^{1}H NMR spectrum presented in Figure S2 on the aromatic region (BPO/toluene-d_8).

2. Analysis of BPO decay kinetics

We conducted NMR experiments in toluene-d_8 at different concentrations of BPO to investigate its decomposition kinetics without SWCNTs. Having deciphered the NMR spectra of neat BPO, we were able to measure the decay kinetics of BPO at two concentrations, i.e. 3.8 mM (Figures S4-S5) and 7.5 mM (Figures S6-S7). Every sample was measured before and after heating for 30, 90 and 180 minutes at 100°C.

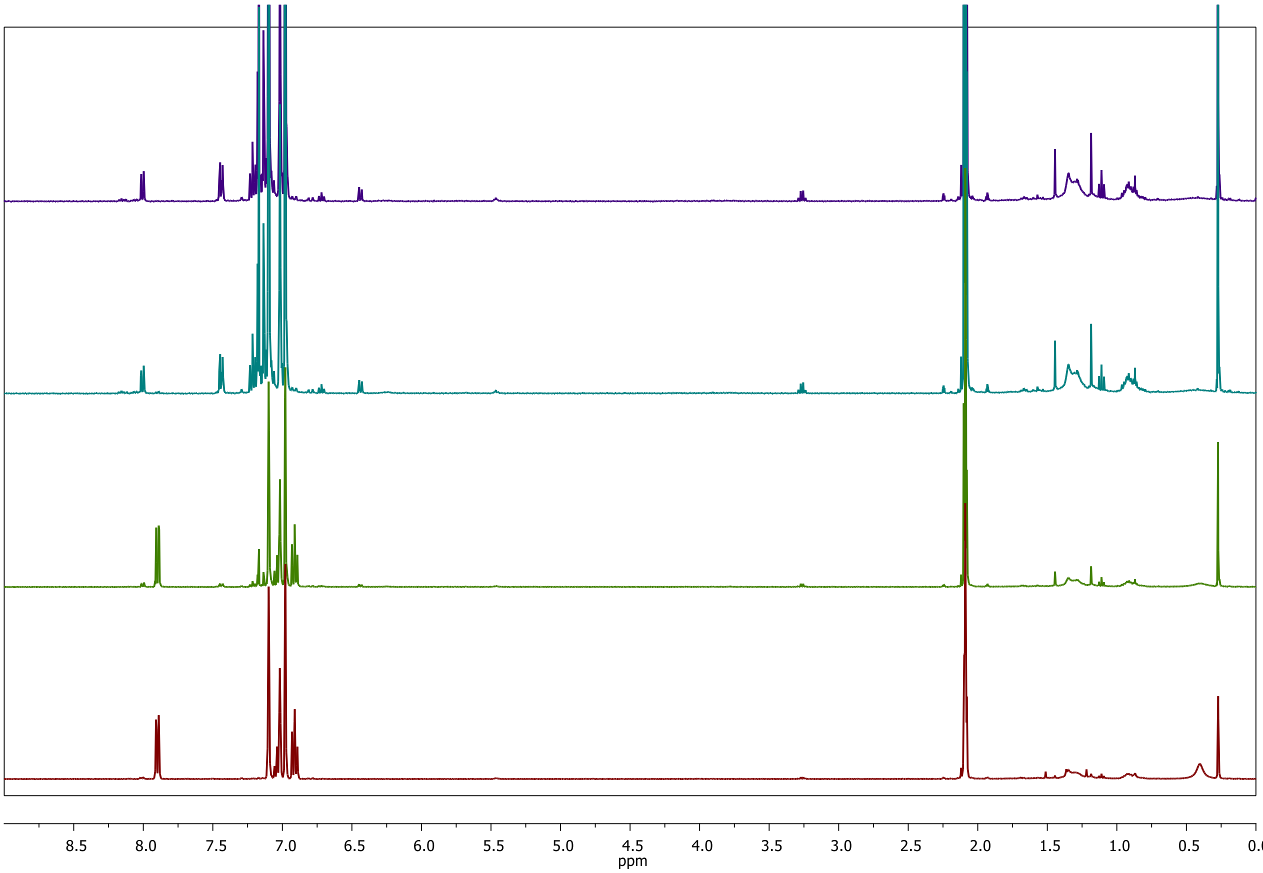

Figure S4 Full ^{1}H NMR spectra of BPO (3.8 mM) dissolved in toluene-d_8 and heated for 0 min (reference - red), 30 (green), 90 (turquoise) and 180 minutes (violet) at 100°C.

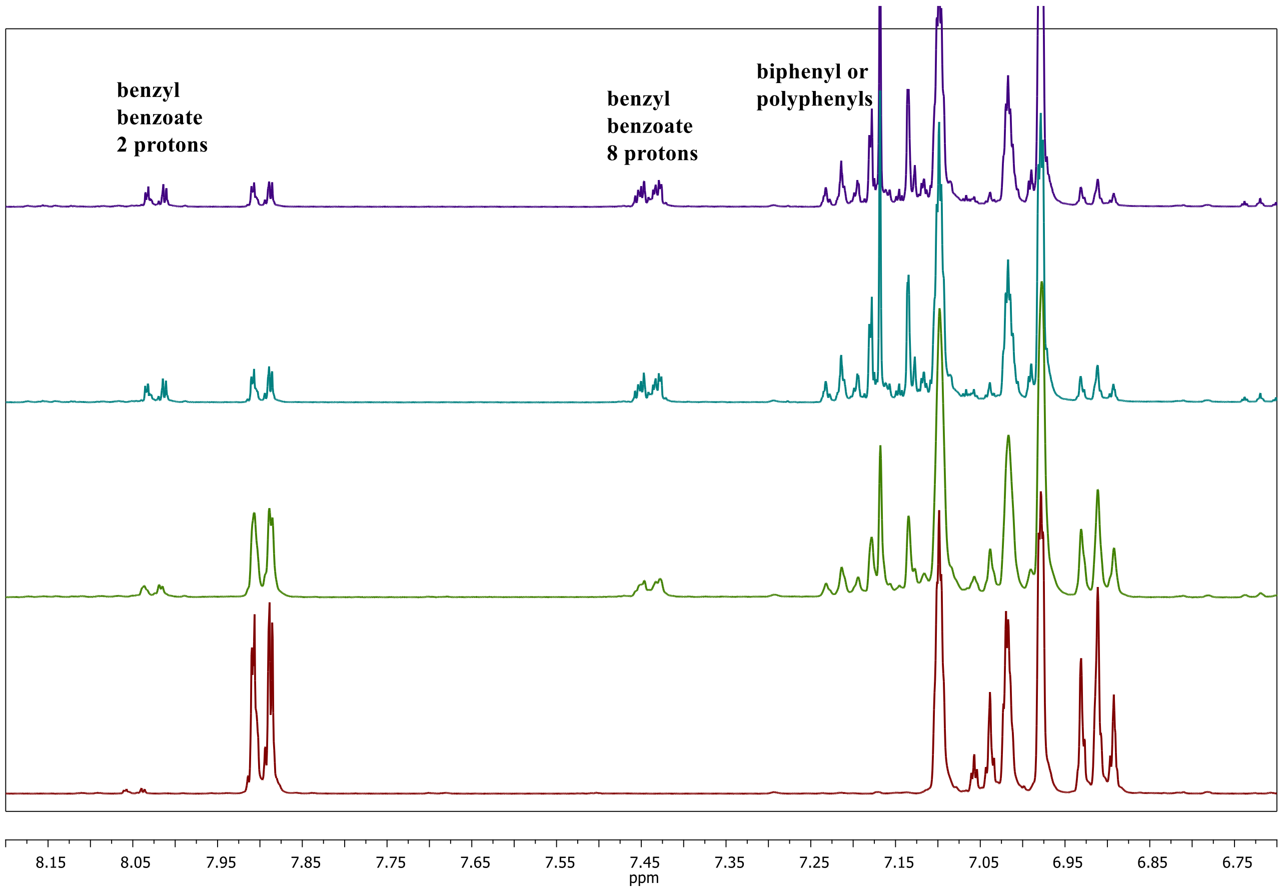

Figure S5 Magnification of the ^{1}H NMR spectra presented in Figure S4 on the aromatic region of BPO (3.8 mM) dissolved in toluene-d_8 and heated for 0 min (reference - red), 30 (green), 90 (turquoise) and 180 minutes (violet) at 100°C. Signals from decomposition products were assigned according to AIST SDBS database (https://sdbs.db.aist.go.jp/) i.e. SDBS No :7302 Spectral code: HSP-02-127 for benzyl benzoate; SDBS No :1182 Spectral code: HSP-40-249 for biphenyl.

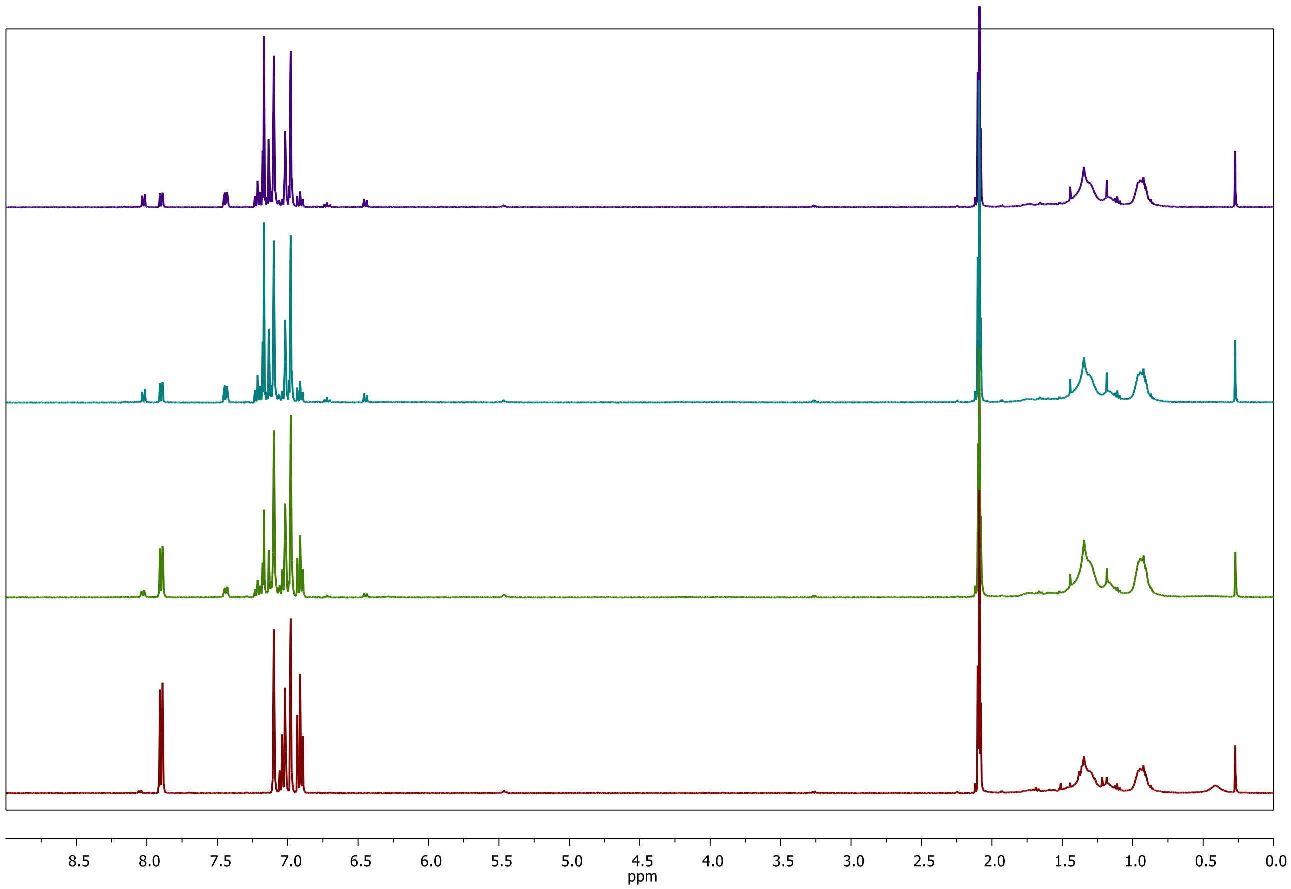

Figure S6 Full ^{1}H NMR spectra of BPO (7.5 mM) dissolved in toluene-d_8 and heated for 0 min (reference - red), 30 (green), 90 (turquoise) and 180 minutes (violet) at 100°C.

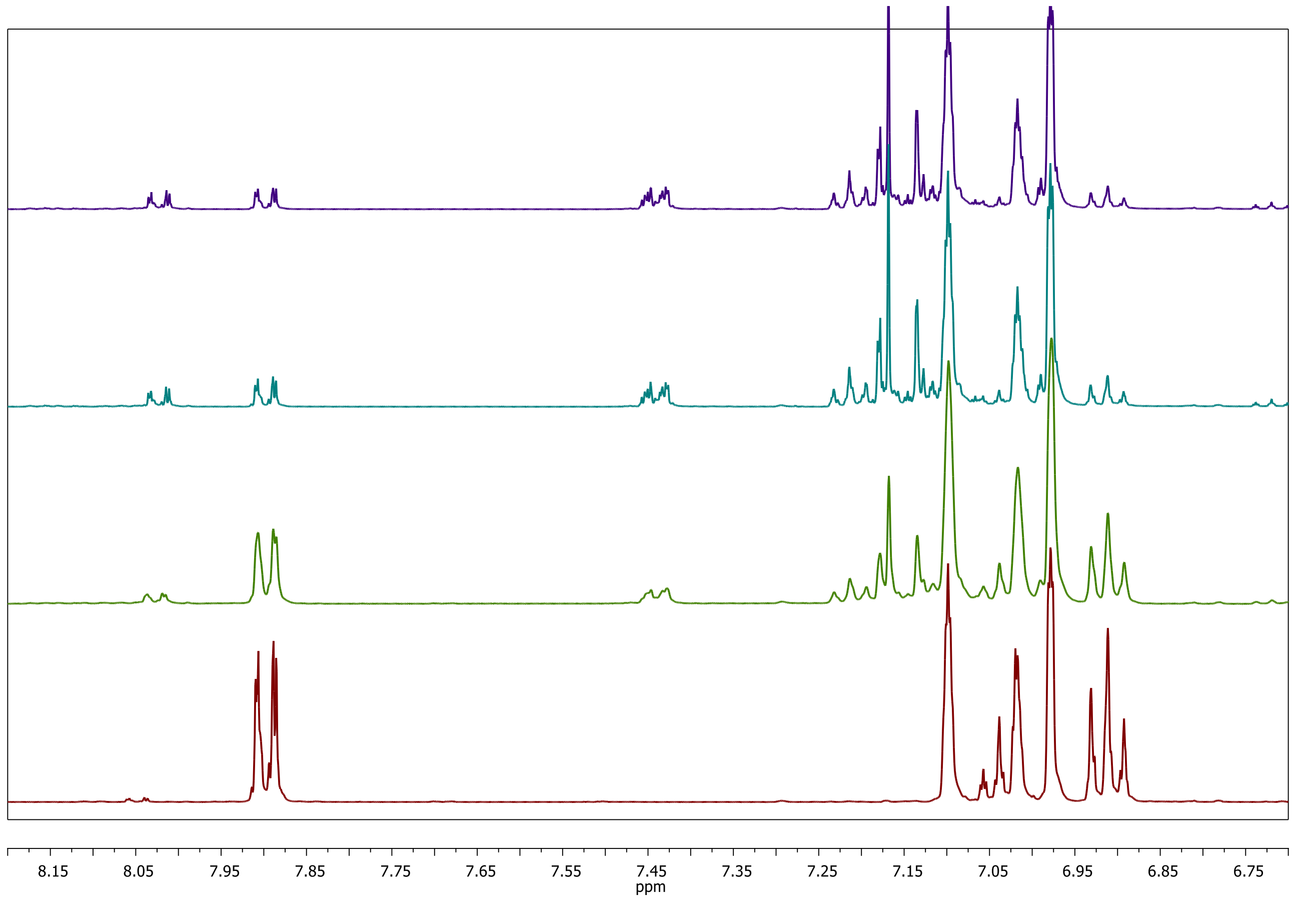

Figure S7 Magnification of the ^{1}H NMR spectra presented in Figure S8 of BPO (7.5 mM) dissolved in toluene-d_8 and heated for 0 min (reference - red), 30 (green), 90 (turquoise) and 180 minutes (violet) at 100°C.

Our analysis of the decomposition products (Figures S4-S7), corroborated by literature studies [4], indicates that the decomposition of BPO primarily yields biphenyl, benzyl, and phenyl benzoate, along with trace amounts of benzoic acid. From the obtained spectra, the relative amounts of BPO (based on signals at 7.9 or 6.9 ppm), toluene-d_8 (7.1 or 6.98 ppm), and benzyl benzoate (8 ppm), can be measured with high accuracy. This provides a method for monitoring the kinetics of the process by comparing the changes in the ratio of signals from BPO (after subtracting the protons from toluene) to the sum of signals from the decomposition products. To perform quantification, the spectra were integrated, and the signals were assigned to BPO and the products of its decomposition. Consequently, BPO : residue ratios were calculated.

Table S1. Ratios of ^{1}H NMR signals of BPO to signals from the products of its degradation.

Time [min]	BPO concentration	
	**3.8 mM**	**7.5 mM**
0	4.840	3.7
30	0.375	0.65
90	0.108	0.018
180	0.089	0.006

We plotted the obtained values as a function of time and fitted the data with an exponential decay function. No significant change was observed between the decomposition kinetics of BPO for 3.8 and 7.5 mM concentrations (Figure S8). Hence, the decomposition profile of BPO in toluene-d_8 does not seem to depend on the BPO concentration to a statistically significant extent.

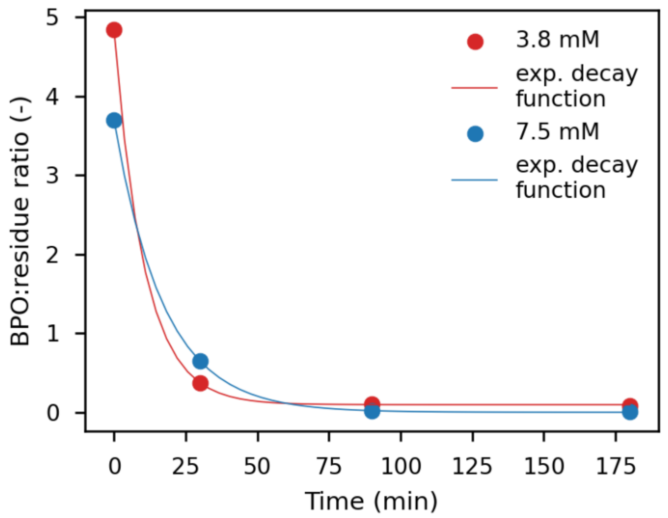

Figure S8 The kinetics of BPO decomposition monitored by ^{1}H NMR, by comparing the ratios of BPO signals to signals from its decomposition products.

3. Analysis of interactions between PFO-BPy6,6' and generated radicals

To investigate the reactivity of BPO toward the polymer, we performed additional ^{1}H NMR experiments. The first was to confirm the thermal stability of pure polymer in toluene-d_8, which was heated at 100°C, for 30, 90, and 180 minutes.

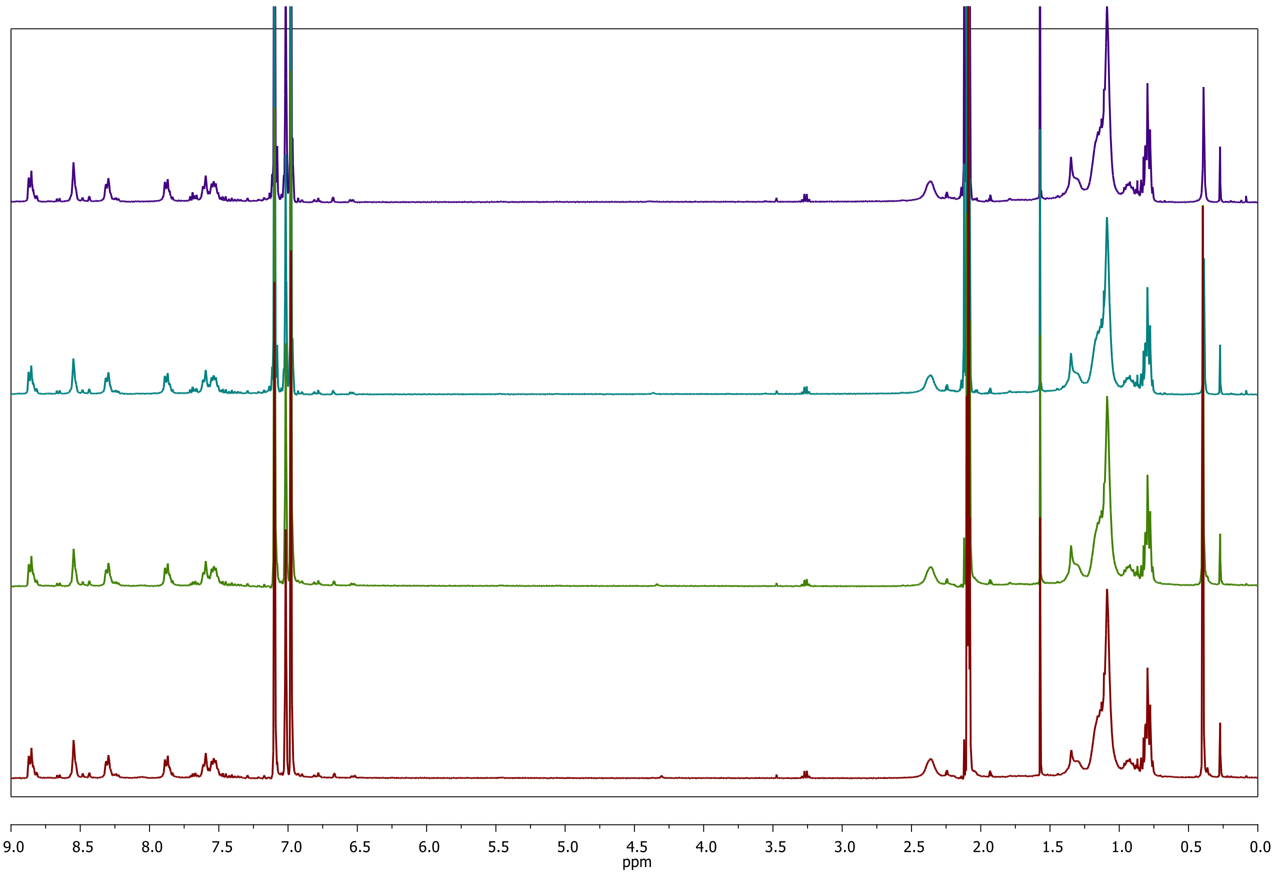

Figure S9 ^{1}H NMR spectra of PFO-BPy6,6' dissolved in toluene-d_8 without heating (red) and after heating at 100°C for 30 min (green), 90 min (turquoise), and 180 min (purple). The signals are consistent with the published NMR spectra for this material [5].

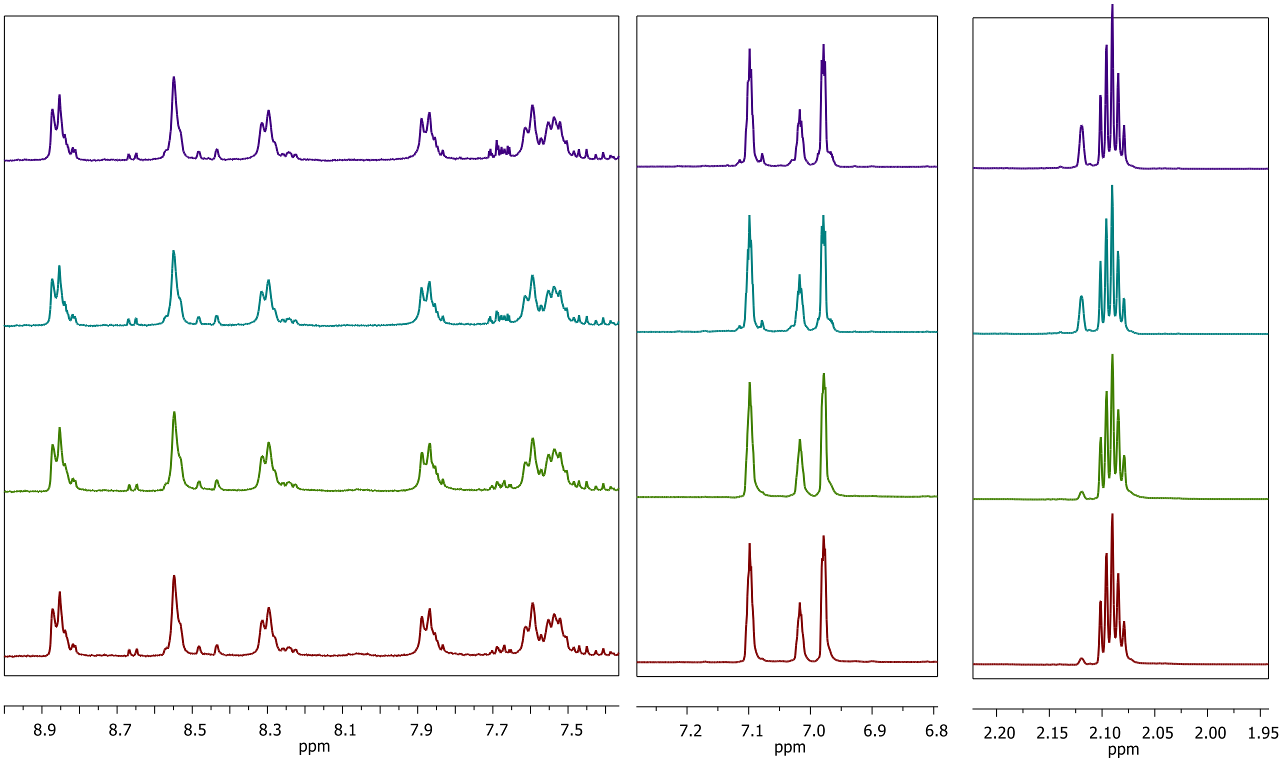

Figure S10 ^{1}H NMR spectra of PFO-BPy6,6' dissolved in toluene-d_8 without heating (red) and after heating at 100°C for 30 min (green), 90 min (turquoise), 180 min (purple). The signal assignments are consistent with the NMRs published in our previous work [5].

The spectra of the polymer remained unchanged after the treatments, indicating its thermal stability. Analogously, BPO alone was subjected to such processing to see how its spectrum changes when heated and what new signals emerge from its decomposition products (Figures S11-S13).

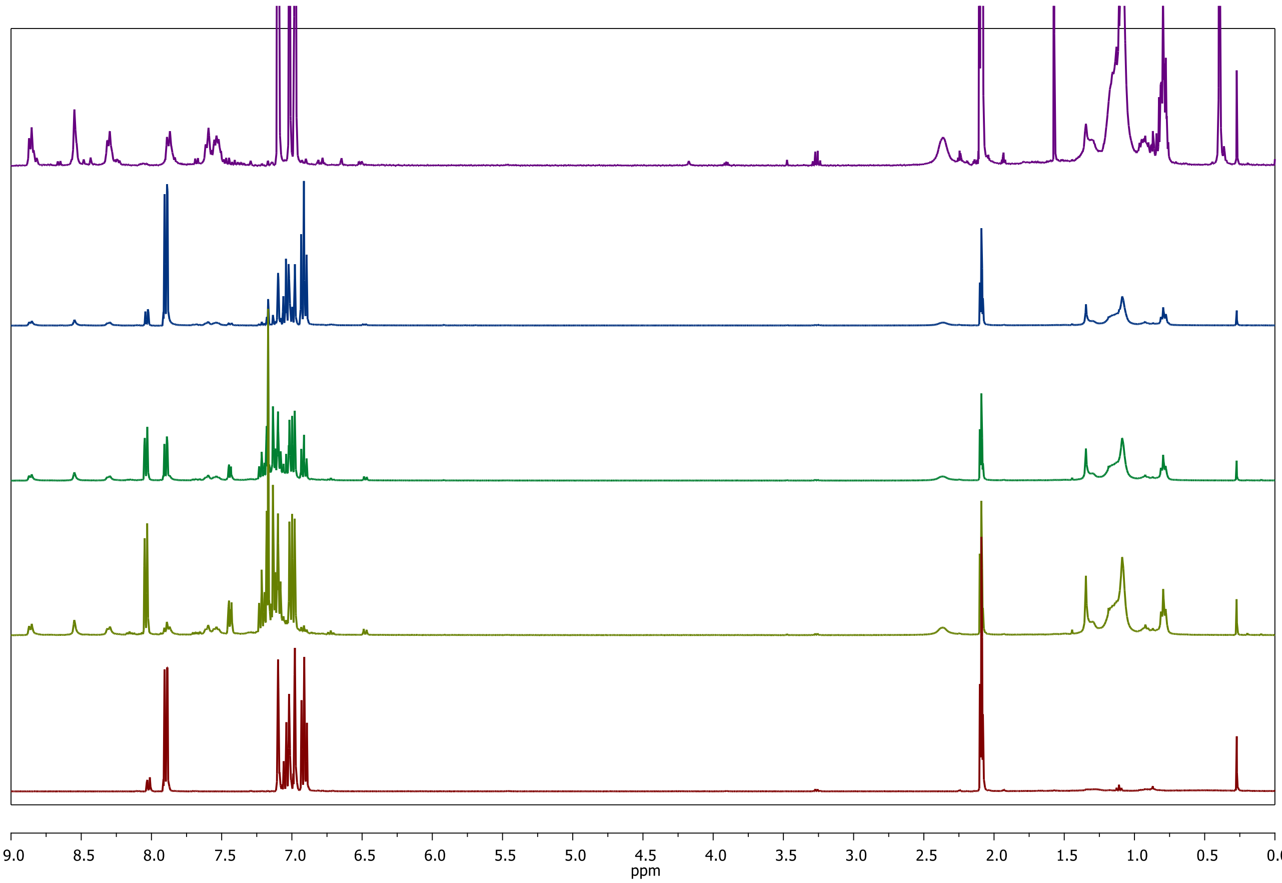

Figure S11 Full ^{1}H NMR spectra of BPO dissolved in toluene-d_8 without heating (red) and after heating at 100°C for 30 min (light green) and 180 min (green). For comparison, the spectra of a mixture of PFO-BPy6,6' with BPO heated 30 min at 100°C (blue) and the polymer alone heated 30 min at 100°C (purple) are also shown.

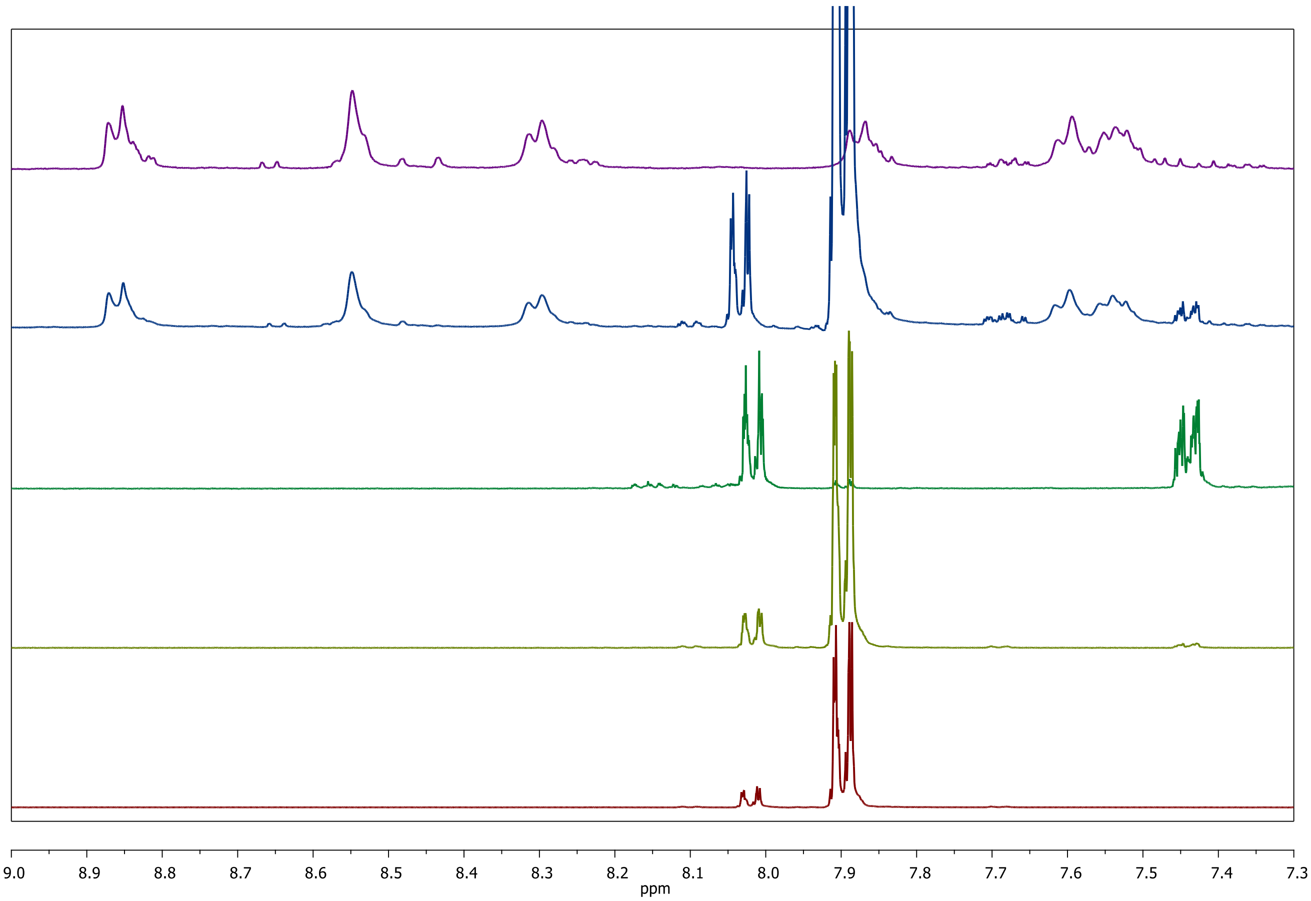

Figure S12 Magnification of the aromatic region of the ^{1}H NMR spectra of BPO dissolved in toluene-d_8 without heating (red) and after heating at 100°C for 30 min (light green) and 180 min (green). For comparison, the spectra of a mixture of PFO-BPy6,6' with BPO heated 30 min at 100°C (blue) and the polymer alone heated 30 min at 100°C (purple) are also shown.

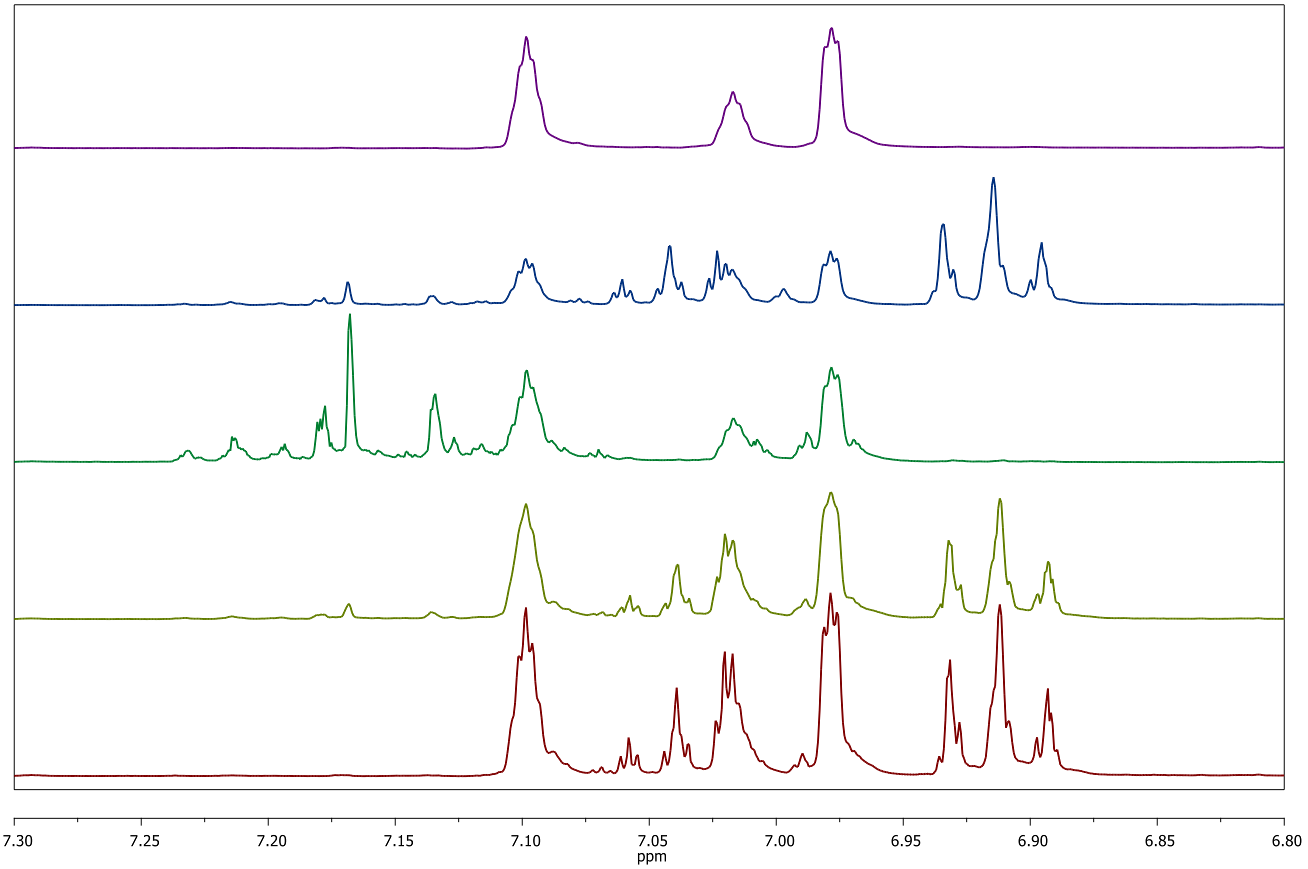

Figure S13 Magnification of another aromatic region of the ^{1}H NMR spectra of BPO dissolved in toluene-d_8 without heating (red) and after heating at 100°C for 30 min (light green) and 180 min (green). For comparison, the spectra of a mixture of PFO-BPy6,6' with BPO heated 30 min at 100°C (blue) and the polymer alone heated 30 min at 100°C (purple) are also shown.

Comparing the compiled spectra, no relevant changes were observed to the chemical identity of the polymer in the presence or absence of BPO. However, it should be considered that the analysis of the potential functionalization of the polymer by means of NMR is difficult due to the limited solubility of the PFO-BPy6,6' polymer in toluene (solubility values are around 1 to 2 mg/mL depending on the molecular weight of the polymer) [1]. Therefore, the intensity of signals from the polymer is limited and usually much lower than analogous signals from BPO, which shows no solubility problems. In summary, direct comparison of the spectra of the polymer and the polymer/BPO mixture (before and after heating) did not provide evidence supporting the possibility of PFO-BPy functionalization by BPO. If it occurred, the functionalization effect was negligible and stayed below the threshold of detection of NMR spectroscopy.

4. Determination of water content via Karl Fischer titration

To determine the water content in the samples, a Metrohm 915 KF Ti-Touch Karl Fischer titrator was utilized. We conducted Karl Fischer titration measurements to accurately determine the water content in various samples: non-dried toluene, toluene dried with 4 Å molecular sieves (used in our experiments), a dispersion of (6,5) SWCNTs in toluene, commercial BPO at a concentration of 5 mM (tested without drying, after drying with anhydrous $MgSO_4$, and after drying with 4 Å molecular sieves), commercial BPO at 15 mM concentration (as received and with an additional 25 µL of water added), and finally, in-house synthesized BPO at 15 mM concentration.

Table S2 Results of water content determination via Karl Fisher titration.

Name	Desiccant	Average residual water content (ppm)
Toluene	None, „wet" commercial solvent	212.8 ppm
Toluene	Freshly dried with 4 Å molecular sieves	19.9 ppm
SWCNT dispersion in toluene	None	126.8 ppm
BPO (comm.), 5 mM	None, 25% wetted commercial reagent	316.5 ppm
BPO (comm.), 5 mM	Dried with anhydrous $MgSO_4$	186.6 ppm
BPO (comm.), 5 mM	Dried with 4 Å molecular sieves	166.4 ppm
BPO (comm.), 15 mM	None, 25% wetted commercial reagent	1070.5 ppm
BPO (comm.), 15 mM	None, 25% wetted commercial reagent with additionally supplied 25 µL of water	2123.4 ppm
BPO (synth.), 15 mM	None, on-site synthesized	112.3 ppm

Our measurements confirmed high water content in the commercial BPO and demonstrated the effectiveness of the employed drying procedures. Additionally, the deliberate addition of water to the system significantly increased the water content in the reaction mixture. To compare the functionalization process under conditions with minimal water content, we utilized the on-site synthesized and dried BPO to reduce residual moisture. Our drying procedure, incorporated into the workup phase of BPO synthesis, was inspired by protocols for the synthesis of asymmetric peroxides using the m-CPBA reagent[6]. This method involves dissolving BPO in a neutral solvent (e.g., chloroform or dichloromethane), followed by drying over anhydrous magnesium sulfate ($MgSO_4$). This approach effectively removes water and reduces the risk of decomposition during long-term storage with drying agents.

5. Characterization of (6,5) SWCNT dispersion before and after chemical modification

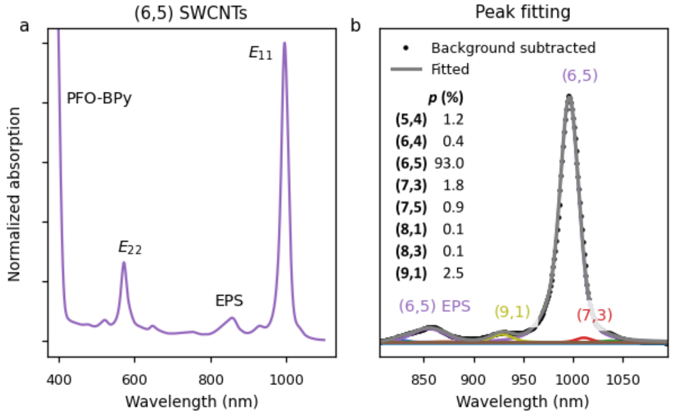

Figure S14 a) Normalized optical absorption spectrum of (6,5) SWCNTs showing E_{11} and E_{22} electronic transitions and exciton-phonon sideband (EPS). The peak near 400 nm originates from PFO-BPy used for sorting SWCNTs. b) Deconvolution of the optical absorption spectrum from panel (a). Peak fitting was used to determine chiral purity *p* for every SWCNT chirality present in the dispersion (details are provided in the Experimental section).

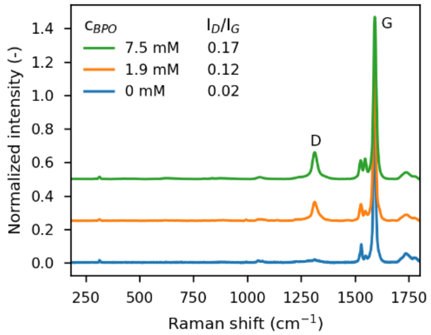

Figure S15 Normalized Raman spectra of pure (6,5) SWCNTs and subjected to 1.9 mM and 7.5 mM BPO treatments at 100°C.

To obtain Raman spectra, 3 mL of a sample containing chirality-enriched SWCNT suspension (with E_{11} absorption in the range of 0.6-0.8) was filtered through a PTFE membrane (Ahlstrom, 0.22 µm pore size) under reduced pressure. The SWCNTs on the membrane were then washed with 5 mL of hot toluene (80 °C) and redispersed in 200-300 µL of fresh toluene. The material was subsequently drop-casted on a glass substrate for characterization. The samples were measured using Renishaw inVia spectrometer with 532 nm laser.

6. The influence of the relative amount of (6,5) SWCNT with respect to BPO on the course and extent of SWCNT functionalization at 100°C

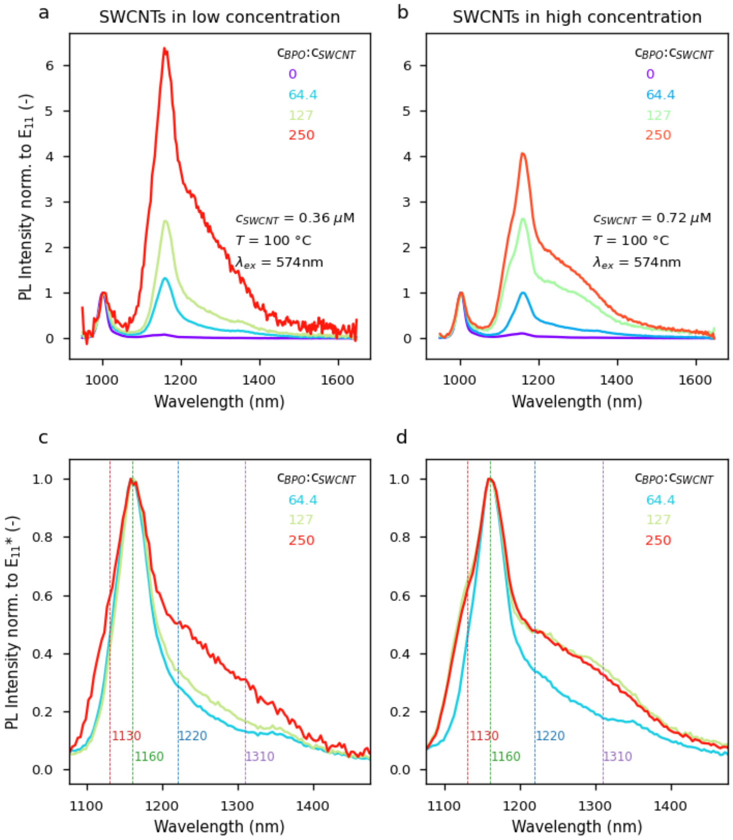

Figure S16 PL spectra of low (0.36 µg/mL) and high concentration (0.72 µg/mL) of (6,5) SWCNTs functionalized with BPO at 100 °C, using different initial molar ratios of BPO:SWCNTs in the range from 0 to 250.

7. Functionalization of (6,5) SWCNT dispersion with BPO at 70°C and 100°C

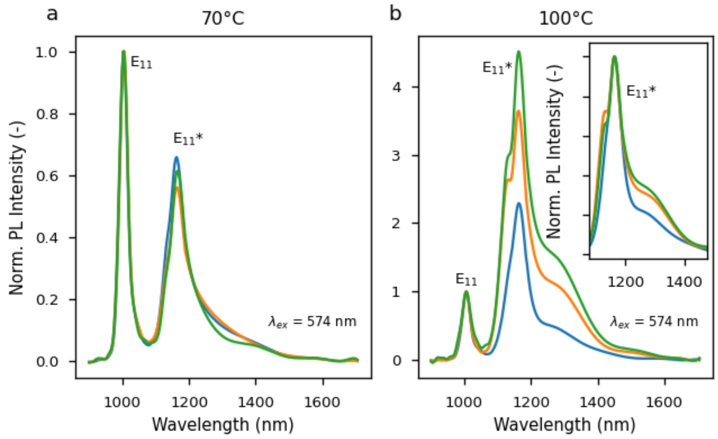

Figure S17 PL spectra of (6,5) SWCNTs functionalized with 3.8 mM BPO at a) 70 and b) 100°C for 1 h. The preparation conditions were identical in the case of all three samples presented in a given panel. The variability in results observed at 100°C is caused by variations in the induction periods of the BPO decomposition in organic solvents, leading to different degrees of SWCNT functionalization after one hour, even when comparing identically prepared samples. The inset shows data normalized to the E_{11}* intensity for spectral shape comparison.

8. Kinetics of (6,5) SWCNT dispersion functionalization with BPO at 100°C

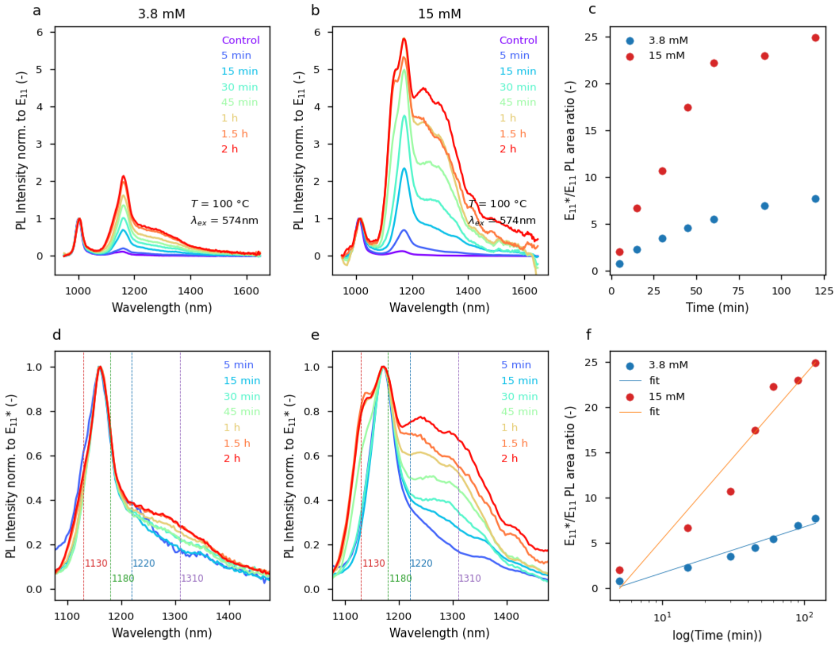

Figure S18 PL spectra of (6,5) SWCNTs functionalized using a,c) 3.8 and b,d) 15 mM as a function of time. In c) and d) are normalized to E_{11}* intensity value for comparison of spectral variability. e) The E_{11}*/E_{11} ratios of the integrated intensities of these peaks as a function of time. f) logarithmic relationship between the reaction time and the E_{11}*/E_{11} ratios noted by linear fitting.

9. The influence of processing conditions on the functionalization of (6,5) SWCNTs

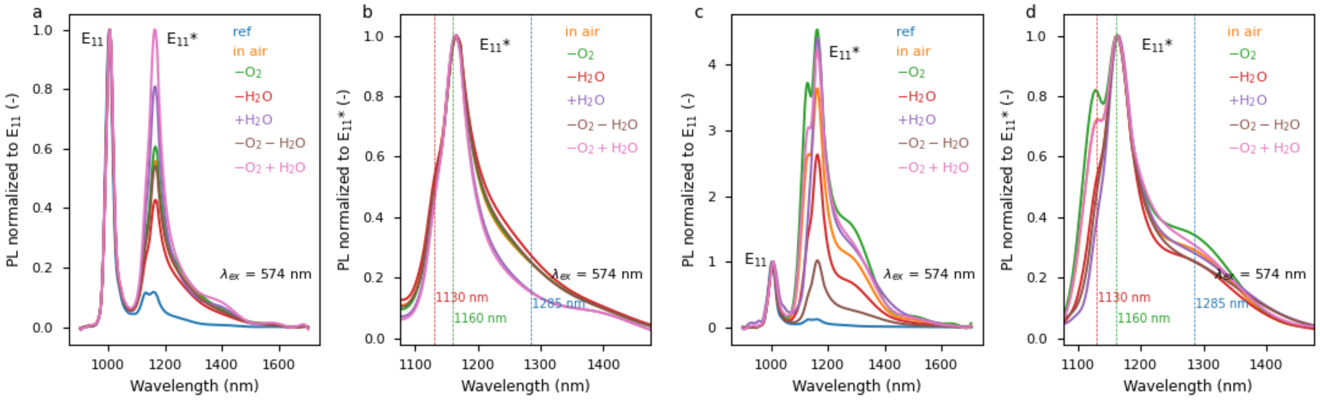

Figure S19 PL spectra of the (6,5) SWCNTs reacted with 3.8 mM BPO at 70°C for an hour, normalized to a) E_{11} for comparison of functionalization degree and b) to E_{11}* for comparison of spectral diversity. c) and d) show the analogous set of spectra for the SWCNTs reacted at 100°C. Functionalization conditions: in the absence of oxygen ("-O_2", flushed with argon), in the absence of water ("-H_2O", dried with molecular sieves), with the intentional addition of water ("+H_2O"), in the absence of oxygen and water ("-O_2-H_2O"), and in the absence of oxygen but with intentional addition of water ("-O_2+H_2O").

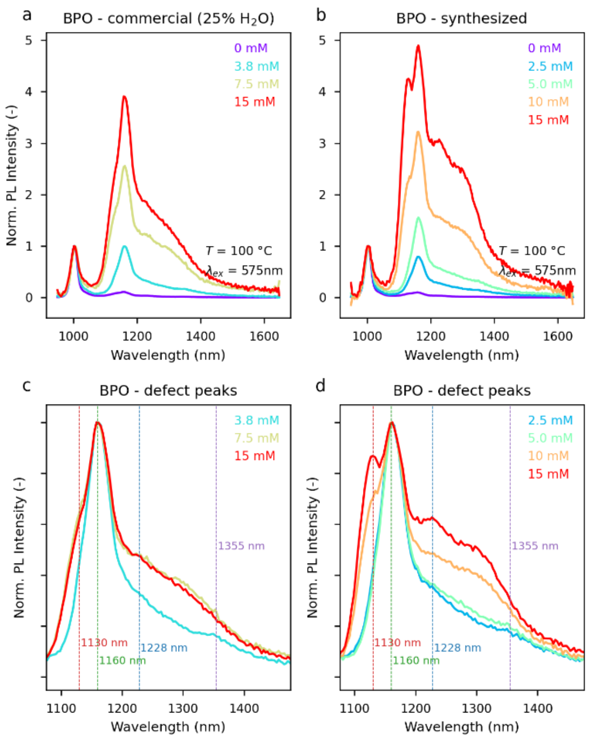

Figure S20 PL spectra of SWCNTs functionalized with commercial (wetted) and in-house synthesized BPO (low moisture content). normalized to (a,b) E_{11}, and (c,d) E_{11}* optical transitions.

To assess the impact of water on the functionalization process, we compared the kinetics of SWCNT functionalization using both the dry, in-house synthesized BPO and the commercial water-containing BPO. The results clearly demonstrated that water presence moderated the functionalization process, leading to reduced

functionalization extent, particularly observed in the 1130 nm and 1200-1220 nm regions of the PL spectra (Figure S19). These findings suggested that water may interfere with radical generation or stabilization, affecting the formation of benzoate radicals and their subsequent attachment to SWCNTs.

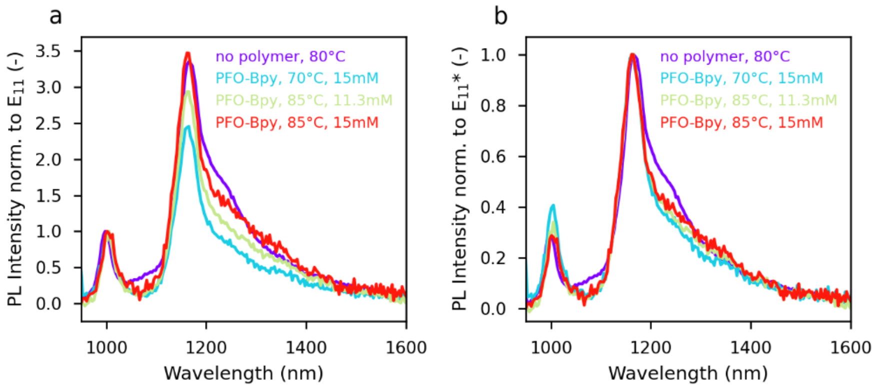

Figure S21 PL characterization of (6,5) SWCNTs functionalized without (purple) and with PFO-BPy6,6 wrapping (red, blue, green) after functionalization under various conditions.

10. Functionalization of SWCNTs in mixed-solvent media

Table S3 Selected properties of co-solvents used for dissolving BPO prior to SWCNT functionalization, sorted in ascending order of dielectric constant [7–11]

Solvent	Density (20-25°C) (g/mL)	Viscosity (20-25°C) (mPa·s)	Dielectric Constant (-)	Dipole Moment (D)	Boiling Point (°C)
hexane	0.661	0.31	1.89	0	69
diphenyl ether	1.070	1.99	1.90	1.17	258
cyclohexane	0.779	0.84	2.02	0	81
decalin	0.896	2.40	2.23	0	192
toluene	0.867	0.56	2.38	0.36	111
o-xylene	0.879	0.76	2.57	0.54	144
tetralin	0.970	2.02	2.77	0.61	207
THF	0.890	0.50	7.40	1.75	66
DCB	1.306	1.07	9.93	2.54	179
acetonitrile	0.786	0.33	37.5	3.92	82
DMSO	1.100	1.99	46.7	3.90	189

Each organic solvent used in this work was characterized by recording absorption spectra to recognize the effect of re-absorption of the SWCNTs' PL by solvent molecules. The normalized spectra in the visible to near-infrared range are shown below (Figure S22). While the peaks overlapped with certain areas of PL emission from SWCNTs, there was no clear correlation between the fluorescence profile of the formed defects and the presence/absence of solvent signals in the particular parts of the spectra.

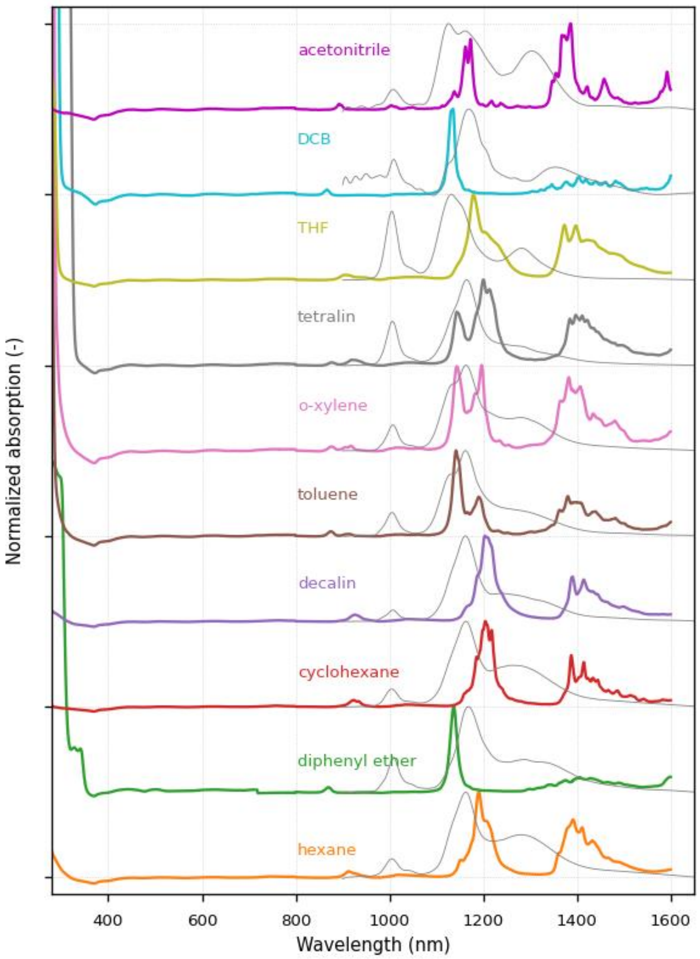

Figure S22 Normalized absorption spectra of various organic solvents used for SWCNT functionalization. Thin grey lines show the PL spectra of BPO-functionalized SWCNTs obtained in the respective solvent (mixed in a 1:1 vol. ratio with toluene).

Further, PL excitation-emission maps were also registered to prove the lack of potential PL signals, which could have come from impurities possibly present even in high purity grade commercial solvents.

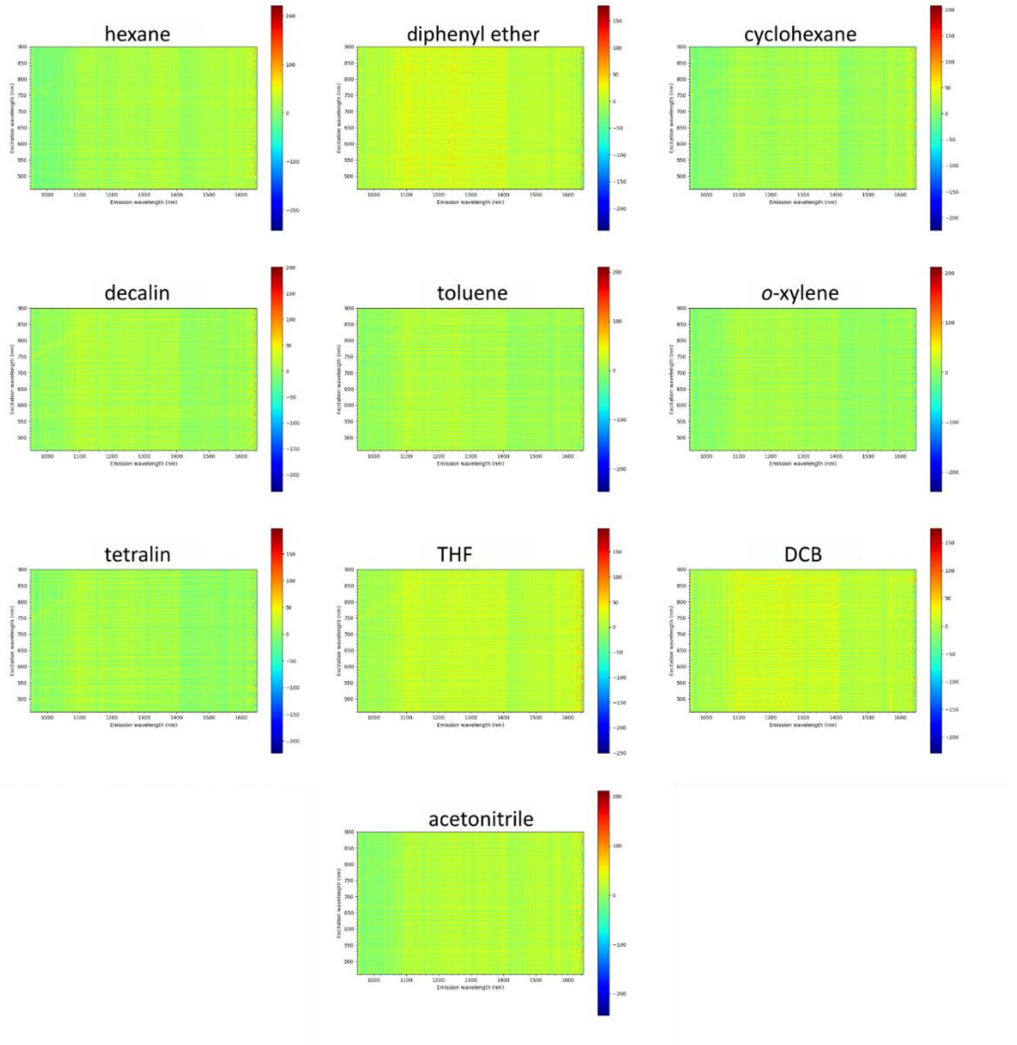

Figure S23 PL excitation-emission maps of the co-solvents used for functionalization of (6,5) SWCNTs showing no emission in the range of measurement.

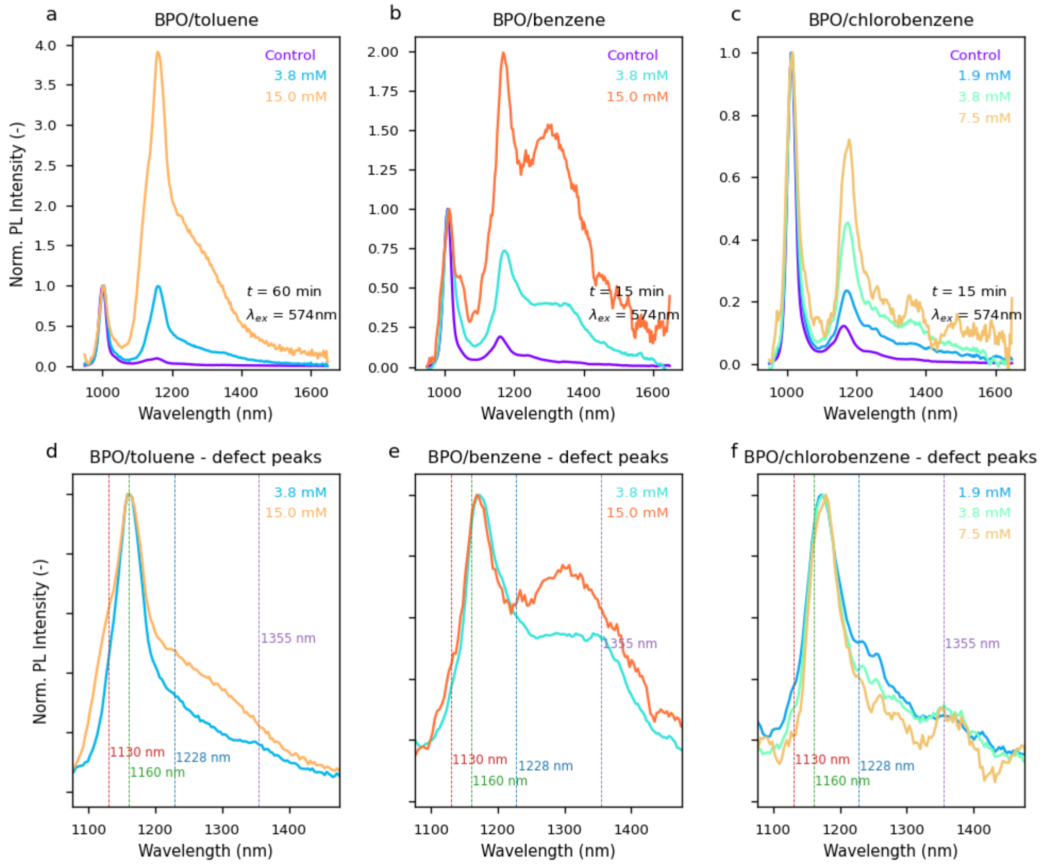

Figure S24 The PL spectra of (6,5) SWCNTs functionalized with BPO at various concentrations in three different solvents: a) toluene, b) benzene, and c) chlorobenzene, normalized to E_{11} intensity. In d), e), and f), defect emissions are compared by normalization to E_{11}^{*} intensities (1160 nm) for a), b), and c), respectively. Dashed lines show the supposed defect peak positions.

11. Literature

[1] A. Dzienia, D. Just, T. Wasiak, K.Z. Milowska, A. Mielańczyk, N. Labedzki, S. Kruss, D. Janas, Size Matters in Conjugated Polymer Chirality-Selective SWCNT Extraction, Advanced Science 11 (2024) 2402176. https://doi.org/10.1002/advs.202402176.

[2] N. Yadav, S.R. Bhatta, J.N. Moorthy, Visible Light-Induced Decomposition of Acyl Peroxides Using Isocyanides: Synthesis of Heteroarenes by Radical Cascade Cyclization, J. Org. Chem. 88 (2023) 5431–5439. https://doi.org/10.1021/acs.joc.2c03059.

[3] W.-Y. Yu, W.N. Sit, Z. Zhou, A.S.-C. Chan, Palladium-Catalyzed Decarboxylative Arylation of C−H Bonds by Aryl Acylperoxides, Org. Lett. 11 (2009) 3174–3177. https://doi.org/10.1021/ol900756g.

[4] A. Yu, N. Zhou, X. Liang, M. Hua, X. Pan, Y. Jiang, J. Jiang, Process hazard and decomposition mechanism of benzoyl peroxide in the presence of incompatible substances, Journal of Molecular Liquids 372 (2023) 121146. https://doi.org/10.1016/j.molliq.2022.121146.

[5] A. Dzienia, D. Just, P. Taborowska, A. Mielanczyk, K.Z. Milowska, S. Yorozuya, S. Naka, T. Shiraki, D. Janas, Mixed-Solvent Engineering as a Way around the Trade-Off between Yield and Purity of (7,3) Single-Walled Carbon Nanotubes Obtained Using Conjugated Polymer Extraction, Small 19 (2023) 2304211. https://doi.org/10.1002/smll.202304211.

[6] W. Xiong, Q. Shi, W.H. Liu, Simple and Practical Conversion of Benzoic Acids to Phenols at Room Temperature, J. Am. Chem. Soc. 144 (2022) 15894–15902. https://doi.org/10.1021/jacs.2c07529.

[7] I.M. Smallwood, Handbook of Organic Solvent Properties, Elsevier, 1996. https://doi.org/10.1016/C2009-0-23646-4.

[8] C.L. Yaws, Thermophysical properties of chemicals and hydrocarbons, William Andrew, Norwich, NY, 2008.

[9] R. Stenutz, Tables, (n.d.). https://www.stenutz.eu/chem/ (accessed May 20, 2024).

[10] S. Murov, Properties of Solvents Used in Organic Chemistry, (n.d.). http://murov.info/orgsolvents.htm (accessed May 20, 2024).

[11] J. Benedict, Dielectric Constant - Flowline Liquid & Solid Level Sensors, Switches & Controllers, Flowline Level Sensor, Transmitter, Switch & Control (2017). https://www.flowline.com/dielectric-constant/ (accessed May 20, 2024).
